# Experimental evolution of gene essentiality in bacteria

**DOI:** 10.1128/mbio.03005-25

**Published:** 2025-10-31

**Authors:** Liang Bao, Zan Zhu, Ahmed Ismail, Bin Zhu, Vysakh Anandan, Marvin Whiteley, Todd Kitten, Ping Xu

**Affiliations:** 1 Department of Oral and Craniofacial Molecular Biology, Philips Institute for Oral Health Research, School of Dentistry, Virginia Commonwealth University 224030, Richmond, Virginia, USA; 2 Massey Cancer Center, Virginia Commonwealth University 6889, Richmond, Virginia, USA; 3 School of Biological Sciences, Center for Microbial Dynamics and Infection, Georgia Institute of Technology 123387, Atlanta, Georgia, USA; Carnegie Mellon University, Pittsburgh, Pennsylvania, USA

**Keywords:** gene essentiality, experimental evolution, spontaneous mutations, whole-genome sequencing, *Streptococcus*, F1Fo-ATPase/V1Vo-ATPase/TrkA1-H1 gene pathway

## Abstract

Essential gene products carry out fundamental cellular activities in interaction with other components. However, the lack of essential gene mutants and appropriate methodologies to link essential gene functions with their partners poses significant challenges. We report here the generation in *Streptococcus sanguinis* strain SK36 of deletion mutants in 32 genes previously identified as essential, with 23 mutants showing extremely slow growth. The 23 genes corresponding to these mutants encode components of diverse pathways, are widely conserved among bacteria, and are essential in many other bacterial species. Whole-genome sequencing of 243 independently evolved populations of these mutants has identified >1,000 spontaneous potential suppressor mutations. Many of these mutations define new gene and pathway relationships, such as F1Fo-ATPase/V1Vo-ATPase/TrkA1-H1, which was demonstrated across multiple *Streptococcus* species. Our findings demonstrate that experimental evolution of slow-growing essential gene mutants provides a powerful strategy to identify compensatory mechanisms and functional interactions, offering new insights into bacterial gene networks.

**IMPORTANCE:**

Essential genes are traditionally considered indispensable for bacterial survival, but how they interact with other cellular processes is not well understood. Here, we deleted essential genes from diverse pathways in *Streptococcus sanguinis* and found that many mutants survived, though with severely impaired growth. When allowed to evolve, these mutants repeatedly acquired spontaneous changes in other genes that restored fitness, uncovering compensatory pathways for disrupted functions. Notably, we identified gene-specific adaptations involving energy metabolism and ion transport, revealing unexpected connections between diverse cellular processes. Because many of these genes are widely conserved, our findings show that even essential functions can be bypassed through alternative routes. This work highlights the evolutionary flexibility of bacterial gene networks and provides a new approach to uncover hidden genetic interactions, offering potential insights into novel antimicrobial strategies.

## INTRODUCTION

All living organisms require certain physical properties and biochemical capacities encoded by “essential” genes to sustain basic cellular activities (1–6). *In vivo*, essential gene products carry out these tasks by interacting with other cellular components. Therefore, the essentiality of genes can also depend on the genetic context provided by the additional genes present (2, 7, 8). Relationships may be characterized by negative epistasis when the essentiality of a gene is dependent on the absence or impairment of another gene’s function, such as synthetic lethality (7, 9) or positive epistasis interactions, where an individual with two genes mutated is fitter than a strain possessing a mutant version of only one of the two genes (7, 10, 11). In contrast to binary classification, it is increasingly acknowledged that essential genes exhibit a quantitative spectrum, displaying a gradient of essentiality (2, 12), and a conditional nature (13). For some essential genes, under optimally controlled conditions, deletion can result in viable, albeit severely compromised individuals (14, 15). The loss of essential genes produces intense selective pressure for the fixation of genomic suppressor mutations in the descendants of the original mutants during subsequent generations (14, 15). Furthermore, essential genes can lose their essentiality during long-term evolution and acquire novel functions (16–18). For instance, unlike the essential characteristics of the critical cell cycle regulator *CDK1* in yeasts and animals (19), plant PSTAIRE-type cyclin-dependent kinases, known as *CDKAs* (plant homologs of *CDK1*), play a role in environmental responses that is independent of the cell cycle (16), likely after the acquisition of the plant-specific cyclin-dependent kinases, *CDKBs* (20).

Identifying genes that interact with essential genes can produce many potential benefits, both from a theoretical perspective, such as understanding the basic principles of cellular life (21, 22), and from a practical standpoint, such as addressing various challenges in synthetic biology (23) and combating drug resistance in agents of infectious disease (22). Experimental evolution imposes a natural selective pressure on a population to redirect the evolution toward fitness-improving phenotypes in a controlled laboratory setting (14, 15, 24–30). This methodology has been applied to identify spontaneous mutations associated with fitness-improving phenotypes, such as multicellularity (24), novel metabolic capacity (25), restoration of flagellar motility (31), and fitness of minimal cells (26). Recently, experimental evolution using mutants deleted of essential genes in the yeast *Saccharomyces cerevisiae* revealed a rapid growth phenotype after multiple passages (14, 15). However, aneuploidy of chromosomes was prevalent among evolved populations deleted of essential genes, which hindered the identification of causal suppressors (14, 15). Here, we have established a transformation system to knock out numerous essential genes, including *obgE* and subunits of F1Fo-ATPase genes, all of which have been identified as essential in most bacterial species, including all species of *Streptococcus* examined (http://www.essentialgene.org/). Therefore, most of these mutants represent the first of their kind in any *Streptococcus*. For the 23 essential genes whose deletion resulted in a slow-growth phenotype, we characterized mutants using experimental evolution. By allowing for short-term adaptation, we identified >1,000 spontaneous potential suppressor mutations in 243 evolved populations deleted of essential genes, most of which are substitutions, deletions, and insertions. This feature enabled us to map virtually all mutations to distinct genomic segments of individual open reading frames (ORFs) or intergenic regions. In particular, we showed that the *f1fo* genes were functionally connected to the *v1vo* and *trkA1-trkH1* genes in multiple *Streptococcus* species.

## RESULTS

### Isolating essential gene mutants and analysis of short-term evolution

The genome of *Streptococcus sanguinis* SK36 comprises over 2,000 ORFs (32), of which 218 have been experimentally identified as essential for the organism’s survival when cells are grown on a rich medium—brain heart infusion (BHI) broth or plates—under microaerobic conditions (1). To isolate viable essential-gene deletion mutants, we have modified our transformation procedure to minimize stress (see Materials and Methods). Although we still employed homologous recombination (Fig. S1a), we replaced our conventional 1-hour aerobic transformation with an anaerobic incubation and a duration of up to 24 hours (1). Given oxygen-dependent H_2_O_2_ production by *S. sanguinis* (33), we hypothesized that exposure to oxygen produces a general stress for *S. sanguinis*, and by eliminating this stress, we might be able to obtain mutants that would otherwise be too sick to survive. We tested our hypothesis at the beginning of this project and found it to be correct for at least some of the mutants, and so we continued this practice. Additionally, we optimized the selection conditions by covering the transformants with a thin layer of agar medium and extending the selection period for the transformants to 4–6 days, while retaining anaerobic conditions (see Materials and Methods).

**Fig 1:**
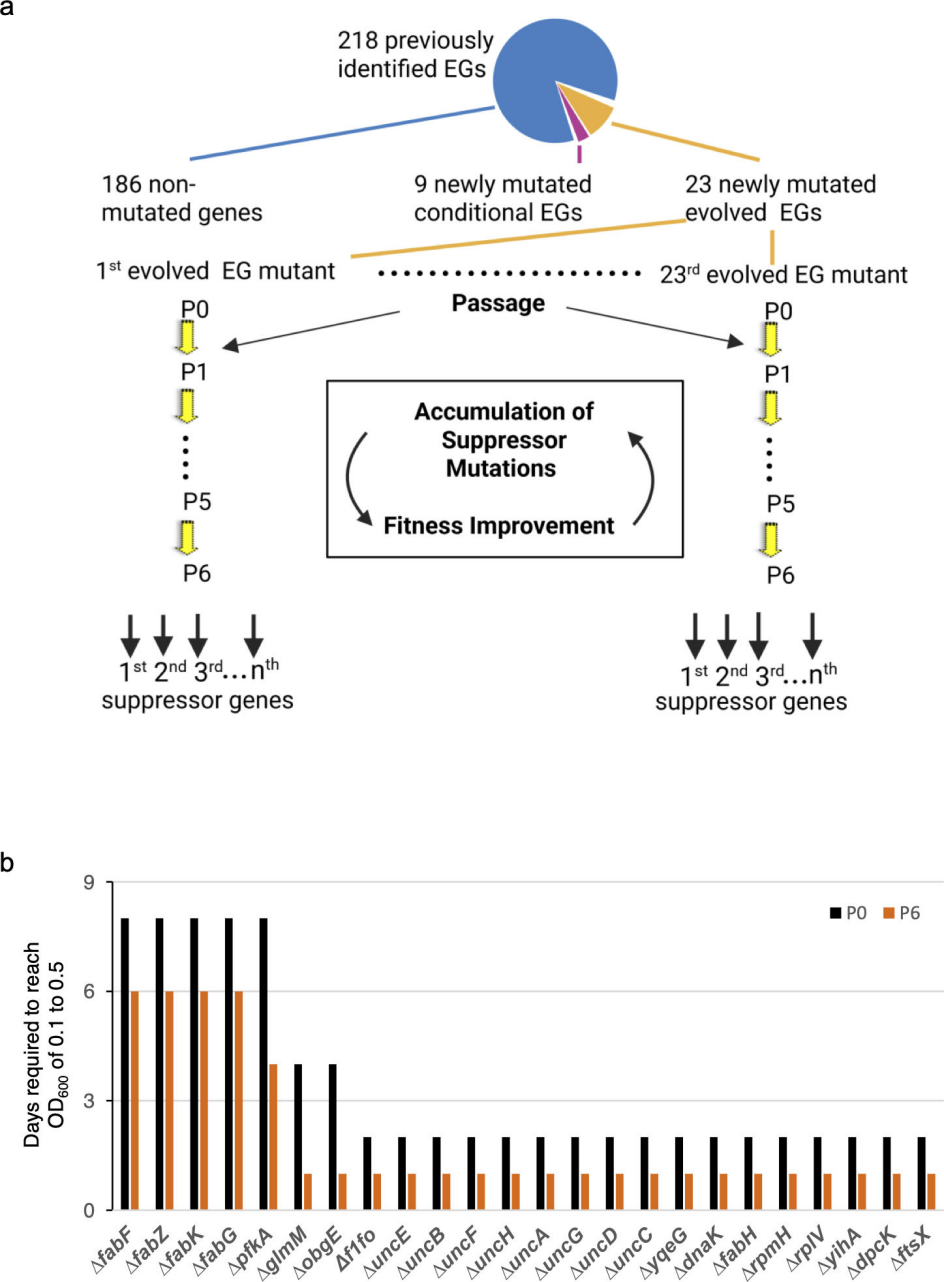
Evolution of mutants deleted for genes previously identified as essential during passage experiments. (**a**) The previously identified 218 essential genes (EGs) were categorized into three groups: 186 genes that we could not delete in this study, nine newly deleted conditional essential genes, and 23 newly deleted essential genes. Mutants deleted for each of the 23 genes in the last group were subjected to experimental evolution, whereby the essential gene deletion mutants were passaged six times to identify suppressor mutations that emerged from this process. Yellow arrows indicate the passaging process. Black arrows indicate the suppressor genes that arose in populations deleted of the newly mutated essential genes. EGs indicate the abbreviation for essential genes. (**b**) Incubation time (days) required for P0 and P6 mutants to reach an OD_600_ of 0.1 to 0.5 in over 50% of populations.

Using this procedure, we observed an interesting phenomenon. Specifically, for 23 of the essential genes (Fig. 1a), the transformants produced two types of colonies: large and small (Fig. S1b). As expected, genotyping of the small colonies revealed a complete replacement of the corresponding ORF with a kanamycin resistance (*kan*) gene (Fig. S1c). In contrast, genotyping of the large colonies showed that 15 of 54 mutants displayed a “double-band” genotype due to duplication of the target gene prior to the replacement of one copy by the *kan* gene (Fig. S1d; Table S1). This finding suggests that *S. sanguinis* SK36 regularly undergoes gene duplications, particularly between two copies of a multi-copy gene or locus, such as the rRNA operons. The remaining 39 large colonies did not contain the *kan* gene, but 38% or 97.4% contained mutations in *fusA*, *rpsL*, or *rplF* (Table S1), which may confer resistance to kanamycin (34). There were also nine essential genes whose deletion mutants had the expected genotype and yet exhibited robust growth (Fig. S2b). These nine genes are *nrdE*, *nrdF*, *nrdH*, and *nrdI*, which were previously reported as non-essential under anaerobic conditions but essential in the presence of O_2_ (35), along with *rexB* for ATP-dependent nuclease subunit B, *rpsA* for 30S ribosomal protein S1, *pdf* for peptide deformylase, *rnaJ* for ribonuclease J, and *g6pD* for glucose-6-phosphate dehydrogenase (Fig. 1a; Fig. S2b).

We therefore directed our attention to the 23 essential genes that, when deleted, led to severe growth defects in the resultant mutants, denoted as newly mutated evolved essential genes (Fig. 1a) (14, 15). These 23 genes encoded components of diverse pathways, including the eight subunit genes encoding the F1Fo-ATPase (*uncE*, *uncB*, *uncF*, *uncH*, *uncA*, *uncG*, *uncD*, and *uncC*); *ftsX*, encoding a permease-like cell division protein; five *fab* genes (*fabH*, *fabK*, *fabG*, *fabF*, and *fabZ*) that are involved in FAS II; two ribosomal protein genes (*rplV* and *rpmH*); two GTPase genes (*obgE* and *yihA*); *yqeG*, encoding a YqeG-family phosphatase; *pfkA*, encoding 6-phosphofructokinase, involved in glycolysis; *glmM*, encoding phosphoglucosamine mutase; *dpcK* (also called *coaE*), encoding dephospho-CoA kinase (DPCK), involved in CoA synthesis; and *dnaK*, encoding a heat shock protein chaperone. These genes are widely distributed and are essential in various genera and species (http://www.essentialgene.org/) (36). In total, we created 23 slow-growing essential gene mutants, each with an individual gene deletion, along with one mutant deleted for the entire *f1fo* region encoding the eight F1Fo-ATPase subunits, which we have named ∆*f1fo* (Fig. S1 and S2a). Although the deletion mutants of the 23 genes were initially generated under anaerobic conditions in this study, we have confirmed the viability of mutants under both anaerobic (Fig. S3a) and microaerobic (Fig. S3b) conditions, as utilized previously for identification of essential genes in *S. sanguinis* SK36 (1). To further verify that the viability of these 24 gene deletion mutants was not due to the absence of oxygen, we selected three essential gene deletions—*pfkA*, *dpcK*, and the entire *f1fo* region—to recreate under microaerobic conditions. These mutants were chosen because these genes are involved in distinct and unrelated biological pathways. We confirmed that we were able to obtain viable, though extremely slow-growing, target gene deletion mutants under microaerobic conditions in addition to the anaerobic conditions used for the initial mutagenesis (data not shown).

In order to identify any suppressor mutations that might arise in slow-growing essential gene mutants, we conducted passage experiments using multiple independently evolved populations of the 24 slow-growing essential gene mutants. To form a population, we selected three to five colonies from the selection medium, combined them into a single inoculum, and subjected them to passage in BHI by 1:20 serial transfer. We proceeded to the next passage only when the cell cultures had reached an OD_600_ within the range of 0.1–0.5 (Fig. 1b). This range was chosen to achieve one passage per day and because a value of ~0.5 was desired, but some mutants did not grow beyond an OD_600_ of 0.1 after 24 hours of incubation. As a control, wild-type (WT) cells, which reached an OD_600_ of approximately 1.0 within 24 hours of growth, or 2–10 times more cells compared to the mutants in each passage, were passaged daily nine times. While all mutants exhibited significant growth defects compared to WT, we observed variations in their growth phenotypes. Notably, mutants lacking *pfkA*, *fabK*, *fabG*, *fabF*, and *fabZ* displayed the most severe growth defects. In contrast, the growth inhibition of others, including another *fab* mutant, *fabH*, was less severe, as evidenced by the OD_600_ measurements of the evolved mutants (Fig. 1b).

### Identification of suppressor loci in evolved populations

**Fig 2:**
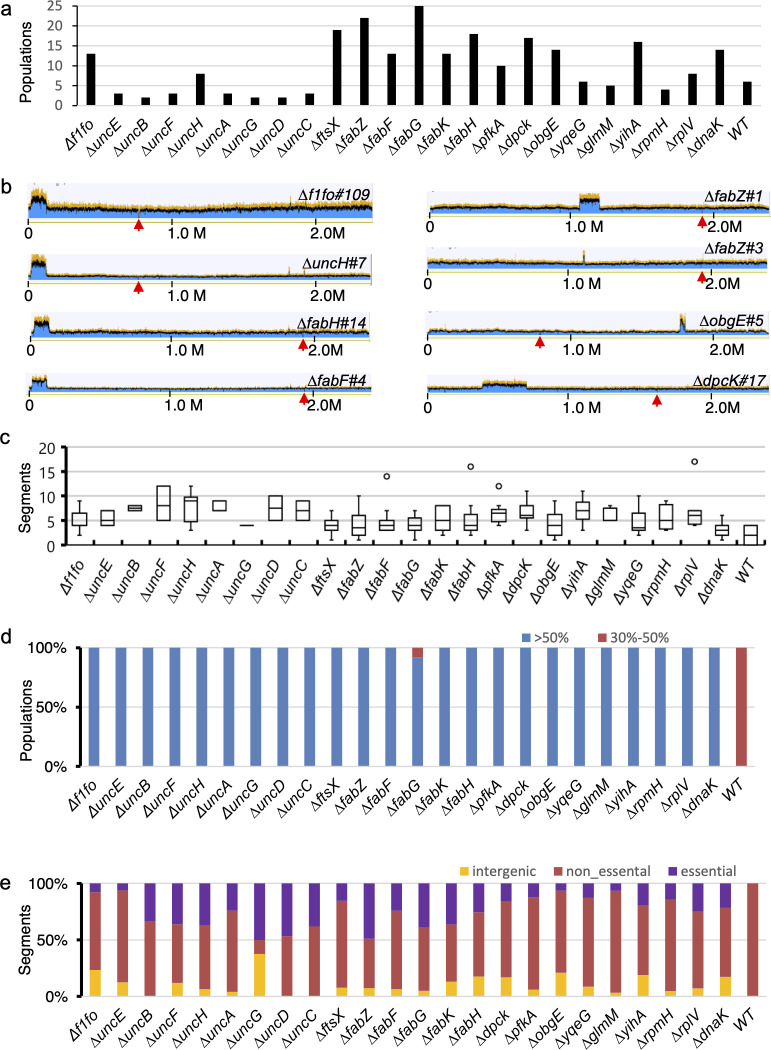
Identification of suppressors in evolved populations. (**a**) The number of independently evolved populations examined for each deletion mutant. (**b**) Evolved populations containing large-scale duplications. Red arrow, site of original essential gene deletion; the height of the blue segments indicates the number of sequence reads mapped to the reference sequence at the coordinates shown on the *X* axis. A doubling of sequence reads indicates a duplication of the affected region. (**c**) Boxplot of mutated segments (≥30% abundance) in the independently evolved populations of mutants deleted for essential genes or WT. Open circles indicate outliers. (**d**) Stacked proportions of populations containing mutated segments comprising >50% (blue) or 30%–50% (red) of sequence reads. (**e**) Stacked proportions of mutated segments belonging to essential ORFs, non-essential ORFs, or intergenic regions.

The genome of *S. sanguinis* can be divided into 4,283 segments—2,340 ORFs and 1,943 intergenic regions (37). We defined a segment as mutated if any mutation within that segment was present in at least 30% of the sequence reads (see Materials and Methods section). We selected 30% as the threshold based on our finding that confirmed suppressor mutations, such as *trkA1-H1*, ranged from 34.8% to 100% in the evolved ∆*f1fo* populations in P6 (Table S2). To identify potential suppressor mutations in the evolved populations deleted of essential genes, we performed whole-genome sequencing at P6. Specifically, we sequenced between 2 and 25 independently evolved populations for each of the 24 slow-growing essential gene mutants, resulting in a total of 243 populations (Fig. 2a; Table S2). As controls, we also sequenced six populations of passaged WT, which were generated by aliquoting into six separate tubes and passaging nine times, as well as the original WT (Fig. 2a; Table S2). The passaged WT cells contained a total of twelve mutations in 10 distinct segments, with five segments specific to WT and five shared with populations derived from the slow-growing essential gene mutants. Notably, the ORF SSA_1920, annotated as phosphotransferase system (PTS) mannose/fructose/sorbose transporter family subunit IID, exhibited mutations in two of the six evolved WT populations, and the ORF SSA_1919, annotated as PTS mannose/fructose/sorbose transporter family subunit IIC, exhibited mutations in one of the populations (Table S2). This result is consistent with a previous study in SK36, where fitness improvement was associated with mutations in three ORFs of the same operon (SSA_1918, SSA_1919, and SSA_1920) (38). In the current study, mutations in these three ORFs were also observed in mutant populations of ∆*uncH*, ∆*fabG*, ∆*fabK*, ∆*fabF*, ∆*fabZ*, and ∆*pfkA* (Fig. S4a and b; Table S2). Interestingly, most of the evolved populations that contain mutations in the three PTS mannose/fructose/sorbose transporter subunits are also those that showed the most severe growth defects, such as ∆*fabG*, ∆*fabK*, ∆*fabF*, ∆*fabZ*, and ∆*pfkA* (Fig. 1b). Importantly, the specific mutations identified in WT populations were never observed in deletion mutant populations, although other distinct *pts* gene mutations were found in the latter (Table S2). Together, these results suggest that mutations in some loci that improved fitness in WT can also promote adaptation in slow-growing essential gene mutants, albeit often through different allelic changes.

We considered first mutations related to duplications of genes other than the essential gene targeted for mutagenesis. Of the 243 evolved mutant populations, we found that 12 (4.9%) displayed gene duplications of various sizes, ranging from 10 kb to 310 kb (Fig. 2b). By comparison, none of the six evolved WT populations in P9 nor the original parental WT contained a gene duplication (data not shown). For the 12 evolved mutant populations with gene duplications, two were from ∆*f1fo* (*#107* and *#109*), one from ∆*uncE* (*#2*), one from ∆*uncH* (*#7*; Fig. 2b; Fig. S5)*,* three from ∆*fabH* (#4, #6, and #14), one from ∆*fabF* (*#4*), two from ∆*fabZ* (*#1 and #3*), one from ∆*obgE* (*#5*), and one from ∆*dpcK* (*#17*; Fig. 2b). The duplicated regions found in populations of ∆*f1fo*, ∆*uncE*, ∆*uncH*, ∆*fabH*, and ∆*fabF* mutants encompassed the same ~103 kb region (coordinate: 22,738–125,834) that is flanked by two directly repeated rRNA operons (Fig. 2b). The above-duplicated region of ~103 kb contains 113 ORFs, including nine ORFs encoding the entire V1Vo-ATPase operon that is related to the proton pump function of F1Fo-ATPase (39), one ORF encoding acyl carrier protein and one ORF encoding an acyltransferase that is involved in lipid biosynthesis (40). The duplicated region found in ∆*fabZ#1* spans approximately 143 kb (coordinate: 1,053,488–1,197,391). The 10.1 kb duplication in ∆*fabZ#3* (coordinate: 1,093,824–1,103,958) is within the duplicated 143 kb in ∆*fabZ#1*. This 10.1 kb region contains 10 ORFs, including one ORF annotated as acyl-acyl carrier protein thioesterase, which catalyzes the terminal reaction of fatty acid biosynthesis (41). In the case of ∆*obgE#5*, a 37 kb duplication is present (coordinate: 1,775,394–1,812,805), encompassing 30 ORFs, including one ORF encoding the Der GTPase. In ∆*dpcK#17*, a ~310 kb duplication occurred (coordinate: 385,678–697,581), encompassing 305 ORFs. The flanking sequences of the duplicated regions in ∆*fabZ#1*, ∆*fabZ#3*, ∆*obgE#5*, and *∆dpcK#17* possessed no notable repeated sequences.

Next, we assessed substitutions, deletions, and insertions of less than 10 kb in the evolved populations. There were six mutations (two substitution mutations and four deletion mutations) from the six evolved populations, where two to four ORFs were affected by each mutation. For example, the C to T mutation (at coordinate 1,313,563) in ∆*fabF#10* caused amino substitutions of two overlapping ORFs encoding two hypothetical proteins (Table S2). The remaining mutations fell within distinct ORFs or intergenic regions. In total, we detected 1,272 mutated segments (Table S2), with 1,260 from evolved populations lacking essential genes and twelve from the evolved WT populations (Table S2). Our analysis revealed that each of the 243 (100%) evolved mutant populations contained at least one mutated segment, with the majority containing from 2 to 10 (Fig. 2c; Tables S2 and S4). In contrast, among the six evolved WT populations, four had at least one segment containing a mutation in at least 30% of the sequence reads, while the remaining two, as well as the parental WT population, had none (Fig. 2c; Table S2). Furthermore, when we set the threshold to 50%, 241 evolved mutant populations, or 99.2%, had mutated segments, whereas none of the six evolved WT populations contained mutated segments (Fig. 2d; Tables S4 and S5; χ^2^ = 154.69, df = 1, *n* = 249 populations, *P* = 2.2 × 10^−16^).

We then analyzed the presence of mutated segments encoding essential genes (in each case, excluding the ORF that was intentionally deleted in each mutant). We found that mutations of essential segments were present in at least one evolved population from each slow-growing essential gene mutant (Fig. 2e; Table S6). In contrast, none of the 12 mutated segments from the evolved WT populations were found to be within an essential ORF (Fig. 2e; Table S7 (*χ*^2^ = 5.74, df = 1, *n* = 25 groups, *P* = 0.017).

In summary, in contrast to the evolved WT populations, mutants deleted for essential genes evolve gene duplications of greater than 10 kb and mutations in essential genes.

### Identification of *v1vo* and *trkA1-H1* mutations in *∆f1fo* mutants

#### Potential suppressor mutations in *∆f1fo*

F1Fo has been proposed to function as an ATP-dependent proton pump in streptococci, including *S. mutans* and *S. sanguinis* (42). Mutations in *trkA1* or *trkH1* are present in all 13 evolved populations of ∆*f1fo*, with 12 populations containing *trkH1* mutations and 1 containing a *trkA1* mutation (Fig. S6a; Table S2). Although TrkA1/H1 mutations reached fixation (>95% of cells) in 7 of the 13 Δ*f1fo* populations, all 13 populations contained more than 30% of cells harboring a TrkA1/H1 mutation. Similarly, mutations in *trkA1*, *trkH1*, or a one base pair (bp) insertion mutation located 38 bp upstream of the *trkA1-H1* operon are found in 25 out of 26 (96.2%) of the evolved populations deleted of single F1Fo subunits (Fig. S6b; Table S2). Among the 24 mutations in the adjacent *trkA1* and *trkH1* ORFs, 16 populations have *trkH1* mutations, and 8 have *trkA1* mutations. Given the comparable length of *trkA1* (1,350 bp) and *trkH1* (1,440 bp), it appears that mutations in the *trkH1* gene are more likely than mutations in *trkA1* to improve the fitness of mutants deleted for F_1_F_o_ subunits.

We also observed that gene duplications occurred in the same region across populations deleted of all F1Fo subunits or single subunits, i.e., ∆*f1fo#107* and *#109*, ∆*uncE#2* and ∆*uncH#7* (Fig. 2b; Fig. S5). This region of approximately 103 kb (coordinates: 22,738–125,834) contains the *v1vo* operon encoding the V1Vo-ATPase, which has been proposed to function as a proton pump (39) and may compensate for the loss of *f1fo*. It is interesting to note that ∆*uncH#7* is the only evolved population listed above that lacks mutations in the flanking upstream regions or the ORFs of *trkA1* or *trkH1* (Fig. 2b). More interestingly, among the evolved populations deleted of F_1_F_o_ subunits (13 ∆*f1fo* and 26 mutants deleted for single *unc* subunit genes), ∆*uncE#2* and ∆*uncH#7* are the only two populations that contain amino acid substitution mutations of V-type ATP synthase subunits (Table S2).

Potential suppressor mutations appeared in other F1Fo subunits or intergenic regions of *f1fo* in many evolved populations deleted of a single subunit (Table S2). This result suggests that for the F_1_F_o_ complex, when one subunit is absent, other subunits cannot function properly, and their presence is detrimental to the mutant.

Environmental factors such as oxygen availability may influence the emergence of compensatory mutations. As a facultative anaerobe, *S. sanguinis* can grow in microaerobic environments as well as anaerobic. To test condition-specific effects, we evolved two F1Fo-ATPase deletion mutants that were generated under microaerobic conditions (6% O_2_) by passaging them under the same microaerobic conditions and observed recurrent mutations in the *trkA1/H1* transport system (Table S2), consistent with patterns seen under anaerobic conditions (Table S2). These results indicate that even under microaerobic conditions, similar adaptive mechanisms emerge as the anaerobic conditions, at least for the suppressor mutation of *trkA1/H1* systems. Future studies will be needed to examine a broader set of gene deletions and environmental contexts to better understand how selective pressures shape evolutionary outcomes in essential gene networks.

#### Role of *V1Vo*, *TrkA1-H1*, and *TrkA2-H2* in the fitness of *∆f1fo* mutants

We have chosen for further analysis F1Fo-ATPase, an evolutionarily ancient enzyme complex that is highly conserved across virtually all forms of cellular life (43) and has been reported as essential for every streptococcal species for which results are reported (36).

**Fig 3:**
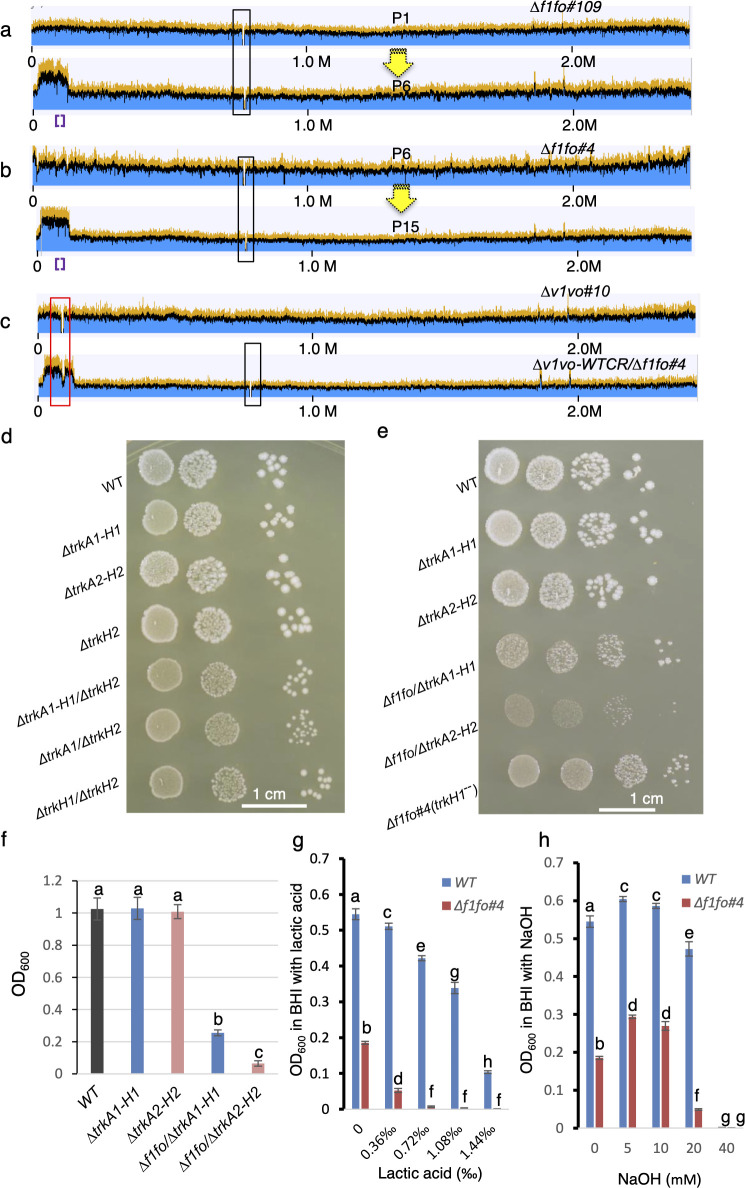
Increased *v1vo* copy number and inhibition of *trkA1-H1* independently contribute to the fitness improvement of evolved Δ*f1fo* populations. (**a**) Gene duplication of the *v1vo* region did not occur in the P1 population (upper) and appeared in the P6 (lower) population of Δ*f1fo#109*. The black box indicates the *f1fo* region. Purple brackets indicate the *v1vo* region. Yellow arrow indicates passaging process. (**b**) Population of Δ*f1fo#4* at P6 that does not contain gene duplication of *v1vo* region (upper). *v1vo* region duplication was observed in P15 (lower). Two out of five evolved populations; see Fig. S7. Purple brackets indicate the *v1vo* region. The yellow arrow indicates the passaging process (**c**) *v1vo* region deleted in WT (upper). Duplication of the *v1vo* region in Δ*f1fo#4,* denoted as *v1voWTCR/*Δ*f1fo#4* (lower, “WTCR” for WT copy retained). The black box indicates the *f1fo* region. (**d**) Growth of strains indicated. Two microliters of cells at an OD_600_ of 0.1 was spotted directly (first column) or diluted 20-fold (column 2), or 400-fold (column 3) and then spotted onto BHI agar and allowed to grow anaerobically for 2 days at 37°C. (**e**) Growth of the strains indicated. Two microliters of cells at an OD_600_ of 0.1 was spotted directly (first column) or diluted 10-fold (column 2), or 100-fold (column 3), or 1,000-fold (column 4) and then spotted onto BHI-agar and allowed to grow anaerobically for 4 days. (**f**) Quantitative growth measurements of WT, *ΔtrkA1-H1*, Δ*trkA2-H2*, Δ*f1fo* in Δ*trkA1-H1* and Δ*f1fo* in *ΔtrkA2-H2*. Cells were cultured anaerobically in BHI for 24 hours. Data are shown as means ± SD from three replicate cultures. Different letters indicate statistically significant differences (*P* ≤ 0.05), determined by one-way analysis of variance (ANOVA) with Tukey multiple comparisons test. Bars that do not share a letter (a, b, or c) indicate samples that are significantly different. (**g**) Effect of lactic acid on the growth of Δ*f1fo#4* and WT. The values are OD_600_ of WT or Δ*f1fo#4* grown in plain BHI or BHI containing different concentrations of lactic acid. The measurements were taken using a Synergy H1 plate reader after 24 hours of growth under anaerobic conditions in 150 µL BHI. *X*-axis denotes the concentration of lactic acid (‰). Data are shown as means ± SD from three replicate cultures. Different letters indicate statistically significant differences (*P* ≤ 0.05), determined by two-way ANOVA with Tukey multiple comparisons test. Bars that do not share a letter indicate samples that are significantly different. (**h**) Effect of NaOH on the growth of Δ*f1fo#4* and WT. The values are OD_600_ of WT or Δ*f1fo#4* grown in plain BHI or BHI containing different concentrations of NaOH. The measurements were taken using a Synergy H1 plate reader after 24 hours of growth under anaerobic conditions in 150 µL BHI. X-axis denotes the concentration of NaOH (mM) in BHI. Data are shown as means ± SD from three replicate cultures. Different letters indicate statistically significant differences (*P* ≤ 0.05), determined by two-way ANOVA with Tukey multiple comparisons test. Bars that do not share a letter indicate samples that are significantly different.

We began by testing whether increasing copy numbers of *v1vo* contributes to the fitness improvement of the mutant deleted for the entire F1Fo complex or single subunits. To explore this, we examined the gene duplication of the *v1vo* region at the early stage, P1, in the four populations that contain gene duplications at P6 (Fig. 2b and 3a; Fig. S5). Among these four populations, one had the gene duplication at P1, while the other three acquired the duplication during subsequent passages (Fig. S5). This observation indicates that gene duplications of the *v1vo* region evolved in these populations after initial passages in three of these four populations.

To further confirm the increased fitness of *v1vo* region duplications in populations lacking *f1fo*, we subcultured five Δ*f1fo#4* populations from the P6 stage, which did not initially contain gene duplications in the *v1vo* region. After nine additional passages (P15), we observed that two out of the five evolved populations had *v1vo* region duplications (Fig. 3b; Fig. S7).

To ascertain the effect of *v1vo* copy number in mutants lacking *fifo*, we tested whether *v1vo* could be deleted in Δ*f1fo*. We first deleted the entire *v1vo*, which consists of nine subunits, to generate the Δ*v1vo* mutant in the WT background. This Δ*v1vo* mutant grew indistinguishably from the wild type in a rich medium (data not shown), demonstrating that *v1vo* can be deleted when *f1fo* is intact. However, when we attempted to delete *v1vo* in Δ*f1fo#4*, where *f1fo* was removed, it resulted in a *v1vo* wild-type copy retained genotype in all five Δ*v1vo/*Δ*f1fo#4* lines tested (Fig. 3c; Fig. S8). Consistent with these findings, ∆*uncE#2* and ∆*uncH#7*, which contain amino acid substitution mutations of V1Vo ATP synthase subunit A and K in the *v1vo* operon, respectively, also contain *v1vo* region duplications (Table S2). This finding indicates that increased copy numbers of the *v1vo* genes compensated for the loss of F1Fo function. Overall, these results suggest that increasing the copy numbers of *v1vo* contributes to fitness improvement of mutants lacking *f1fo*.

We then investigated the impact of loss of *trkA1-H1* on the fitness of Δ*f1fo*. Initially, we compared the occurrence of *trkA1-H1* mutations between P1 and P6 stages across 12 independently evolved populations of Δ*f1fo* (*#101* to *#112*). While mutations in the *trkA1* or *trkH1* segments were not detected in P1 using a 30% threshold in 9 of the 12 populations, we observed *trkA1-H1* mutations in all populations by P6, with the mutation frequency generally increasing during passage (Tables S8 and S9). These mutations produced frameshifts, truncations, and/or amino acid insertions or substitutions in all 12 populations (Tables S8 and S9).

To further investigate the impact of *trkA1-H1*, we compared the growth of Δ*f1fo* mutants in the presence and absence of *trkA1-H1. S. sanguinis* SK36 has two Trk systems, *trkA1-H1* and *trkA2-H2*. The growth of ∆*trkH2* mutants, or double-gene deletion mutants of *trkA1-H1* or *trkA2-H2,* was indistinguishable from that of WT (Fig. 3d). However, combined deletion of either *trkA1* or *trkH1* along with *trkH2* resulted in growth defects (Fig. 3d), indicating that *trkA1-H1* and *trkA2-H2* are redundant for growth. In order to determine the effect of mutating the two Trk systems on the fitness of Δ*f1fo*, we transformed the Δ*f1fo* construct into the backgrounds of Δ*trkA1-H1* or Δ*trkA2-H2* and compared the growth of the resulting strains. As controls, we also included Δ*f1fo#4*, which contains a frameshift mutation of *trkH1* in the Δ*f1fo* background, denoted as Δ*f1fo#4(trkH1^--^*; Table S2). It showed that while the growth of Δ*f1fo#4(trkH1^--^*) and Δ*f1fo/*Δ*trkA1-H1* was comparable to each other, the growth of Δ*f1fo/*Δ*trkA2-H2* was significantly inhibited compared to that of Δ*f1fo#4* Δ*f1fo#4(trkH1^--^*) and Δ*f1fo/*Δ*trkA1-H1* (Fig. 3e and f). These findings suggest that inhibiting *trkA1-H1*, but not *trkA2-H2*, contributes to the fitness improvement of evolved mutants deleted for *f1fo*.

In lactic acid bacteria, F1Fo-ATPase is believed to function by pumping out protons to alkalinize the cytosol or to build negative internal and positive external membrane potentials (44). To test whether the growth defects in the *S. sanguinis* Δ*f1fo* mutant are due to impaired function in alkalinizing the cytosol or building a membrane potential, we compared the growth of Δ*f1fo#4* in a rich medium supplemented with lactic acid (which increases membrane potential and cytosolic proton concentration) or NaOH (which lowers membrane potential and cytosolic proton concentration). We observed that Δ*f1fo#4* is more susceptible to lactic acid treatment than is WT (Fig. 3g). Conversely, the growth defect of Δ*f1fo#4* can be partially rescued by NaOH within a certain range (Fig. 3h). These results suggest that *f1fo* in *S. sanguinis* functions by pumping out protons to alkalinize the cytosol.

### Conserved functional relationships of *f1fo*, *trkA1-H1*, *trkA2-H2*, and *v1vo* in different streptococci

**Fig 4:**
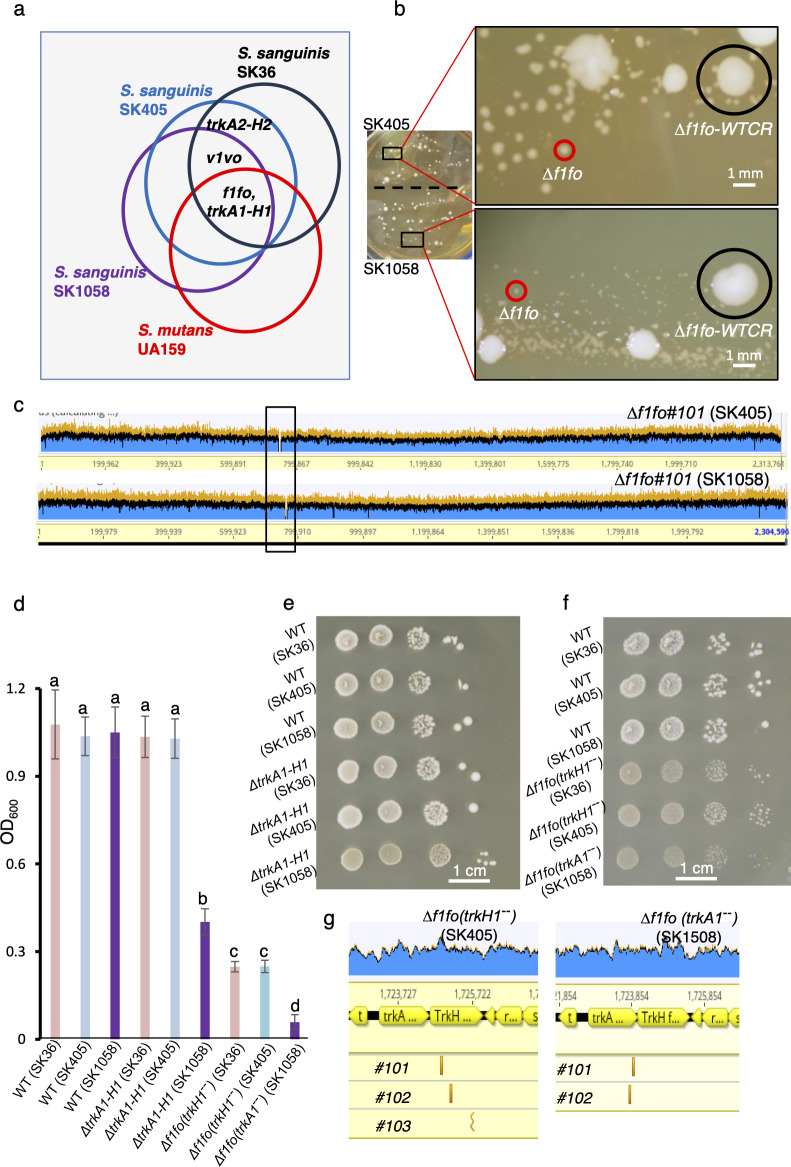
Essentiality of *f1fo* in *S. mutans* UA159, *S. sanguinis* SK36, SK405, and SK1058. (**a**) Venn diagram showing distribution of the genes indicated in *S. mutans* UA159, *S. sanguinis* SK36, SK405, and SK1058. Note that *S. mutans* UA159, *S. sanguinis* SK405, and *S. sanguinis* SK1058 contain both *f1fo* and *trkA1-H1,* as in SK36, while only *S. sanguinis* strains contain *v1vo. TrkA2-H2* is present in both *S. sanguinis* SK36 and SK405 but not in *S. sanguinis* SK1058 or *S. mutans* UA159. (**b**) Growth of *Δf1fo* transformants in *S. sanguinis* SK405 (upper) and SK1058 (lower) on same BHI agar medium for 5 days. Enlarged images of the transformants are shown. Red circles indicate small colonies, *Δf1fo*. Black circles indicate large colonies, Δ*f1fo-WTCR* (WT copy retained). (**c**) Deletion of *f1fo* in SK405 or SK1058 shown by whole-genome sequencing. Black box indicates the location of the *f1fo* region. (**d**) Quantitative measurements of growth in SK36, SK405, or SK1058 WT, Δ*trkA1-H1*, or Δ*f1fo* mutants. Cells were grown in BHI broth for 24 hours. Data are shown as means ± SD from three replicate cultures. Bars with different letters are significantly different from one another (*P* ≤ 0.05), as determined by one-way ANOVA with Tukey multiple comparisons test. (**e**) Growth of SK36, SK405, or SK1058, Δ*trkA1-H1* mutant in the backgrounds of SK36, SK405, or SK1058. Cells at an OD_600_ of 0.1 in a volume of 2 µL were spotted directly (first column) or diluted 10-fold (column 2), or 100-fold (column 3), or 1,000-fold (column 4). Cells were grown on BHI-agar for 2 days anaerobically. (**f**) Growth of SK36, SK405, SK1058, and their Δ*f1fo* mutants. Cells were diluted as in Fig. 4e and grown on BHI-agar for four days anaerobically. (**g**) Location of *trkA1-H1* suppressor mutations in SK405 and SK1058 Δ*f1fo* mutants. Mutations in *trkA1* or *trkH1* appeared in all evolved Δ*f1fo* populations, three in SK405 and two in SK1058. *X*-axis indicates the genome coordinates. Gold bars indicate mismatches between the sequences of the suppressor mutants and the parent (Δ*f1fo*) strains.

To assess F1Fo’s essentiality in other strains and species, we conducted essentiality evaluations in three other streptococci: *S. mutans* UA159, *S. sanguinis* SK405, and *S. sanguinis* SK1058. Genomic sequencing of *S. mutans* UA159 (45), *S. sanguinis* SK405 (37), and *S. sanguinis* SK1058 (37) showed that *f1fo* and *trkA1-H1* are present in all three strains, as in SK36, while *v1vo* is present in the *S. sanguinis* strains only. The *trkA2-H2* genes are present in both *S. sanguinis* SK36 and SK405 but not in *S. sanguinis* SK1058 or *S. mutans* UA159 (Fig. 4a).

To assess whether *v1vo* could serve as a partial backup in case of F1Fo loss-of-function, we attempted to delete *f1fo* in *S. mutans* UA159, which lacks *v1vo*. Despite multiple attempts and a total of 64 independent genotyping analyses, none of the transformants were deleted for *f1fo*, instead producing a double band indicative of gene duplication prior to allelic exchange (data not shown). This result highlights that in *S. mutans* UA159, lacking *v1vo, f1fo* cannot be deleted under our conditions. This observation supports our conclusion that *v1vo* reduces cellular dependence on *f1fo*.

Next, we investigated the possibility of deleting *f1fo* in *S. sanguinis* SK1058 and SK405, both of which contain *v1vo* and *trkA1-H1*. We could generate *f1fo* whole-region deletion mutants (Δ*f1fo*) in both SK1058 and SK405 backgrounds (Fig. 4b and c). Furthermore, the growth of mutants with *f1fo* deletions in SK405 was notably better than in SK1058 (Fig. 4b, d through f).

We then investigated the impact of deleting *trkA1-H1* in *S. sanguinis* SK1058, which possesses only *trkA1-H1*, and *S. sanguinis* SK405, which has both *trkA1-H1* and *trkA2-H2*. Our findings showed that *trkA1-H1* mutants exhibited severe growth defects in the SK1058 background compared to the SK405 background (Fig. 4d and e). This result demonstrates the requirements of the *Trk* system for growth within the species of *S. sanguinis*.

To explore the potential emergence of *trkA1-H1* suppressor mutations during evolution, we conducted passage experiments using independently evolved populations with *f1fo* deletions in SK405 (three populations) and in SK1058 (two populations). It was observed that, after six passages, *trkA1-H1* mutations appeared in all evolved Δ*f1fo* populations in SK405 and SK1058 (Fig. 4e; Table S10). At least one population each in the SK405 and SK1058 backgrounds possesses frameshift or truncation mutations of *trkA1-H1* (Fig. 4g; Table S10). This result underscores the evolutionarily conserved occurrence of *trkA1-H1* suppressor mutations in evolved populations with *f1fo* deletions.

### Working model involving *f1fo*, *v1vo*, *trkA1-H1*, and *trkA2-H2*

**Fig 5:**
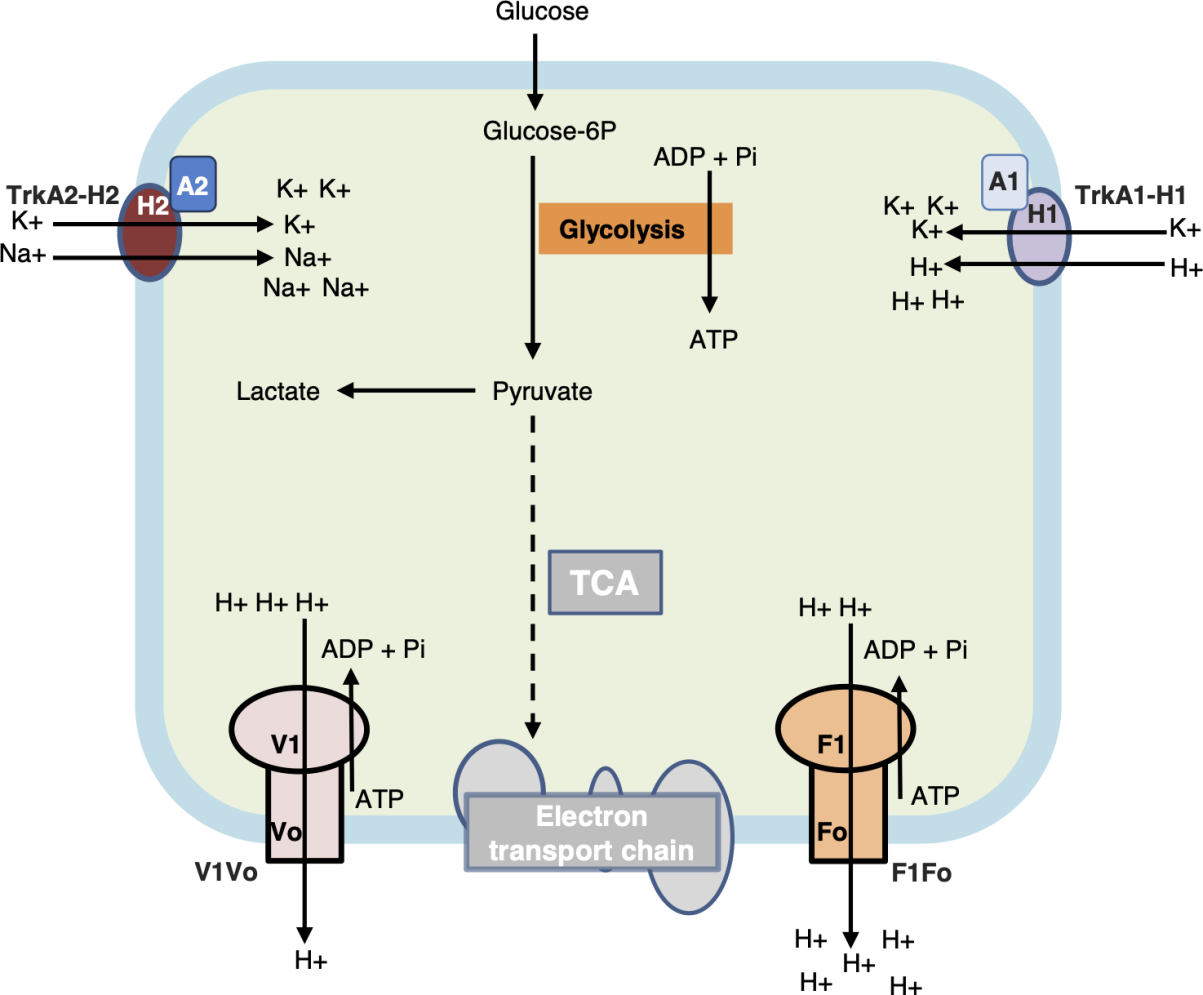
Working model for F1Fo, V1Vo, TrkA1-H1, and TrkA2-H2 relationships. For streptococci, which do not contain a complete tricarboxylic acid cycle (TCA: gray-filled and dashed line) or electron transport chain (ETC: gray-filled), energy production is mainly through lactic acid fermentation. The lactate from fermentation creates acidic conditions in the cytosol, which becomes toxic if H^+^ is not exported. F1Fo pumps out H^+^ from the cytosol using ATP, and V1Vo supplements or replaces the function of F1Fo when the F1Fo is inhibited/inactivated. Both TrkA1-H1 and TrkA2-H1 are potassium transporters and are functionally redundant. TrkA1-H1 pumps in potassium together with H^+^, acidifying the cytosol when H^+^ is not exported. In contrast, TrkA2-H2 does not pump protons into the cytosol when pumping in potassium (likely instead pumping in Na^+^). Therefore, the function of TrkA1-H1 must be inhibited if F1Fo is not functional. Solid lines indicate the presence of reactions. Dashed lines indicate the absence of TCA reactions.

Given that V1Vo may also function as an ATP-dependent proton pump (39), and the Trk systems work as symporters (K^+^/Na^+^ or K^+^/H^+^) (46), we showed that *v1vo* can partially compensate for the loss of *f1fo*, that TrkA1-H1 is likely the major contributor to acidification of the cytosol when pumping in K^+^, and the function of TrkA1-H1 must therefore be inhibited when F1Fo is not present. We therefore suggest the following model for the relationship of *f1fo* with *v1vo*, *trkA1-H1*, and *trkA2-H2* (Fig. 5). For streptococci, which do not contain the tricarboxylic acid cycle or electron transport chain, energy production is mainly through lactic acid fermentation (47). The lactate from fermentation creates a highly acidic cytosol, which would be toxic if H^+^ were not exported. F1Fo pumps out H^+^ from the cytosol using ATP. Both TrkA1-H1 and TrkA2-H2 are potassium transporters and functionally redundant for that purpose. TrkA1-H1 pumps in potassium together with H^+^, acidifying the cytosol when H^+^ is not exported. In contrast, TrkA2-H2 does not pump protons into the cytosol when pumping in potassium (likely instead pumping in Na^+^). Therefore, the function of TrkA1-H1 must be inhibited if F1Fo is not functional.

### Potential suppressor mutations in *∆dpcK*

To further validate the suppressor mutations identified in this study, we investigated a second essential gene, *dpcK*, which encodes DPCK, the terminal enzyme in the five-step pathway of CoA biosynthesis (48).

In evolved ∆*dpcK* populations, we identified mutations upstream of *acpS* (encoding holo-ACP synthase) in 11 of 17 populations (Tables S2 and S7). Holo-ACP synthase catalyzes the covalent attachment of CoA to the acyl carrier protein, a critical step in fatty acid biosynthesis (49).

To characterize these upstream mutations, we recovered 11 ∆*dpcK* populations from frozen stocks and PCR-amplified ~1 kb spanning the 107 bp region upstream of *acpS* for Sanger sequencing, along with the corresponding region from WT (Fig. S9a; see Table S11 for primer sequences). Among the 11 ∆*dpcK* populations, eight carried 42 bp deletions located –17 to –58 bp upstream of the *acpS* start codon, one carried a –91 C→A substitution, and two carried no detectable mutations in this region (Fig. S9a). Notably, the 42 bp deletions consistently occurred between two 6 bp direct repeats (TGTCGG), suggesting a recombination-mediated mechanism (Fig. S9b)

To assess functional consequences of *acpS*-upstream mutations, we compared *acpS* expression among 11 ∆*dpcK* populations and WT. Reverse transcription quantitative polymerase chain reaction (RT-qPCR) analysis revealed that all the eight strains harboring the 42 bp deletion or the lone strain containing the –91 C→A substitution exhibited approximately fourfold higher *acpS* expression relative to wild type and ∆*dpcK* strains lacking these mutations (Table S12), indicating that these upstream changes enhance *acpS* transcription (Fig. S9c).

Interestingly, in one population (∆*dpcK#17*), we also detected a large duplication (>300 kb, encompassing 305 ORFs; Fig. 2b) that included *acpS*. The precise mechanism by which elevated *acpS* expression compensates for loss of *dpcK* remains to be determined.

### Potential suppressor mutations in *∆pfkA*

The *pfkA* gene encodes 6-phosphofructokinase (PFK), responsible for catalyzing the phosphorylation of fructose-6-phosphate to fructose-1,6-bisphosphate, a committing step in glycolysis (50). Among the 10 ∆*pfkA* evolved populations (Tables S2 and S7), 6 populations contained mutations in *fruR*, 5 populations had mutations in *fruB*, and 9 populations had mutations in *glcK*. The *glcK* gene encodes an ROK domain-containing glucokinase, which phosphorylates glucose to produce glucose-6-phosphate, a progenitor of fructose-6-phosphate (51). The *fruR* gene encodes a transcriptional repressor of the *fruBA* operon, while *fruB* (also known as *pfkB*) has been demonstrated to function as a 1-phosphofructokinase in multiple bacterial species (52), including streptococci (53). The *fruA* gene, which is also repressed by FruR (33), encodes a PTS transporter of fructose, which it converts to fructose-1-phosphate concomitant with transport. Interestingly, the nature of the mutations differed according to the gene; 3 of the 11 *glcK* mutations were truncations, and four of the seven *fruR* mutations were truncations or frameshift mutations, while none of the seven *fruB* mutations were truncations or frameshifts (Tables S2 and S7).

We hypothesize that in the absence of *pfkA*, accumulated fructose-6-phosphate becomes toxic, as has been shown previously in other bacterial species (52). Elimination of GlcK activity would reduce fructose-6-phosphate production. We further hypothesize that FruB possesses low levels of fructose-6-phosphate kinase activity, thus replacing PfkA, but only when the expression of *fruB* is increased due to *fruR* mutation, or the fructose-6-phosphate kinase activity of FruB is increased due to alteration of its sequence. Finally, we hypothesize that the addition of glucose to the growth medium should improve growth of WT cells, but not the *pfkA* mutants, while the addition of fructose, which would be transported and converted to fructose-1,6-bisphosphate by the combined actions of FruA and FruB, should support growth of WT cells and *pfkA* mutants.

To test this hypothesis, we recovered four independent ∆*pfkA* mutants carrying mutations in *glcK*, *fruR*, and/or *fruB* (Table S2): #2 (*fruR*, *glcK*), #3 (*glcK*, *fruB*), #6 (*glcK*), and #7 (*fruR*, *glcK*). We then assessed whether fructose could rescue their growth. While the ∆*pfkA* mutants exhibited reduced growth in TY or TY supplemented with 20 mM glucose compared to WT, the addition of 20 mM fructose rescued the growth defect of ∆*pfkA* mutants, as expected (Fig. S10; Table S13). This mechanism would be similar to findings in *E. coli*, where a mutation that increased *fruB* expression was found to suppress the phenotype of ∆*pfkA* mutants (54, 55).

## DISCUSSION

While identifying genes that interact with essential genes or compensate for their loss provides many benefits (21–23), this task remains challenging, primarily due to the unavailability of essential-gene deletion mutants and systematic methodologies to connect the function of essential genes with their partners. Traditional approaches, such as conditional promoter-shutoff (56, 57), antisense RNA knockdowns (58) and CRISPR interference (59, 60), while valuable, may exhibit limitations, including incomplete target gene silencing and suboptimal design efficacy (60), thereby potentially compromising the reliability of essential gene functional analyses. Our study successfully established a system to generate viable mutants with deletions in essential genes (Fig. 1; Fig. S2). These essential gene mutants provide an invaluable resource for dissecting the functions of essential genes. Using these 24 slow-growing essential gene mutants, we identified over 1,000 spontaneous mutations in passage experiments (Fig. 2; Table S2). Parallel evolution—where the same genes were repeatedly mutated across independent populations—was commonly observed (Table S7; Supplemental text). For validation, we focused on potential suppressor mutations that repeatedly arose in mutants deleted for *f1fo*, *dpcK*, or *pfkA,* revealing potential compensatory mechanisms. Specifically, the most frequent mutations occurred in *trkA1-H1* for ∆*f1fo*, upstream of *acpS* in ∆*dpcK*, and in *fruR*, *fruB*, and *glcK* for ∆*pfkA*, suggesting gene-specific adaptation. Although it remains unclear why some previously classified essential genes, such as *rpsA*, showed no apparent growth defect when deleted, we did observe several potential suppressor mutations (Table S2) that warrant further investigation.

As expected, our findings support the notion that mutants with deletions of essential genes face stronger selective pressures. These mutants undergo and retain the following significant evolutionary changes. (i) Gene duplications of greater than 10 kb: Our data revealed that large-scale gene duplications were detected exclusively in mutants deleted of essential genes (Fig. 2b). (ii) Substitutions, insertions, and deletions of less than 10 kb: Small-scale mutations, especially those detected in >50% of sequence reads, were more common in mutants deleted for essential genes (Fig. 2d). (iii) Mutations in essential genes: Mutations in other essential genes were observed exclusively in mutants deleted for an essential gene (Fig. 2d). The suppressor mutations identified in our evolved populations deleted of essential genes may reflect the differential gene essentiality over evolutionary time in the pan-genome (17) and reproducibly changing gene essentiality across independently evolving populations (18). These types of mutations, including duplications, may occur in both mutant and wild-type backgrounds, but selection likely drives their enrichment and fixation in the mutant populations.

In this work, we have demonstrated the efficacy of the methodology developed for generating deletion mutants of numerous essential genes in *S. sanguinis* SK36. Furthermore, we have highlighted the potency of experimental evolution (29, 30) in characterizing mutations in slow-growing essential gene mutants, with a particular focus on *f1fo* in *S. sanguinis*. It would be interesting to test the conservation or divergence of mechanisms governing gene essentiality of other genes, as well as across different *Streptococcus* species and other genera. While extracting valuable information by identifying spontaneous suppressor mutations in populations lacking essential genes is possible, conducting experimental evolution requires caution. First and foremost, the choice of an appropriate organism is crucial. Ideally, the selected organism should have a single chromosome, a small genome size, and a rapid cell division rate. Streptococci represent an ideal candidate in this regard. They possess a single circular genome with a relatively modest size, typically ranging from 2.0 to 3.0 megabases (61). This choice contrasts with a previous study that employed budding yeast with multiple chromosomes (14), where most mutations in populations lacking essential genes were related to changes in ploidy, which could hinder the identification of causal suppressors. On the other hand, the identification of mechanisms of genetic suppression requires caution. For the potential suppressor mutations of low frequency or the substitution mutations which we could not confidently characterize as increasing or decreasing the activity of the gene product, further evidence is needed to understand the mechanisms underlying the suppression phenotype. In our experiments, we used an OD range of 0.1–0.5, which approximately reflects the desired cell density (62, 63), at the end of each transfer, which could introduce variability. However, this range was necessary because certain mutants (e.g., *fabZ*, *fabK*, *fabG*, *fabF*, and *pfkA*) did not grow beyond an OD of 0.1 even after 24 hours. To avoid excluding these strains, we adopted this broader range. Although this approach introduced some variability, it allowed inclusion of all mutants and thereby provided a more comprehensive assessment of fitness differences under the experimental conditions. Notably, despite these constraints, we still observed parallel evolution events, such as mutations in *glcK* and *pfkB* in *pfkA* mutants (Table S7).

In general, essential genes tend to be more conserved and evolutionarily ancient compared to non-essential genes (64). It is intriguing to consider when genes emerge within a biological system over time. The suppressor mutations in essential gene mutants, particularly the loss-of-function mutations, can provide valuable insights into dependency relationships. For instance, the absence of *f1fo* in a system may hinder the acquisition of the *trkA1-H1* system. In cases where essential genes function as a complex, such as *f1fo*, our findings indicate that deletion of one component may result in additional deleterious effects caused by the remaining subunit(s) (Table S2). This suggests that all subunits of the complex need to be integrated into the system, at least for the *f1fo* cases. Moreover, understanding the timelines of gene emergence in a system is also crucial for guiding decisions in synthetic biology, determining which genes, as well as the order of genes, to be introduced into a system.

## MATERIALS AND METHODS

### Strains used and growth conditions

In this study, we primarily utilized *S. sanguinis* strain SK36 (32). *S. sanguinis* SK405 and SK1058 strains were described previously (37). *S. mutans* UA159 was obtained from Ann Progulske-Fox (University of Florida). All bacterial cultures were routinely cultured in a BHI medium at 37°C under anaerobic conditions (0.2% O_2_, 9.9% H_2_, 9.9% CO_2_, and 80% N_2_), with residual oxygen removed using either a palladium catalyst or an anaerobic chamber (Coy Laboratory Products, Inc). For long-term storage, strains were maintained at –80°C. When applicable, microaerobic conditions consisted of 6% O_2_, 7.2% CO_2_, 7.2% H_2_, and 79.6% N_2_.

### Transformation and mutant isolation

To prepare competent cells, *S. sanguinis* was cultured in 1 mL of Todd-Hewitt + HS medium and incubated overnight in anaerobic conditions at 37°C. The following day, a 5 µL aliquot of the overnight culture was added to 1 mL of fresh TS + HS medium and incubated for an additional 3 hours.

For creating knockout constructs, we employed 1 kb of homologous 3' and 5' end sequences flanking the coding sequence and resistance genes, *kan* or *Erm*^r^, in overlapping extension PCR (for primers used, see Table S11) (32). For transformation, we mixed 50–500 ng of PCR product DNA with 200 ng of CSP and 300 µL of competent cells. The reactions continued for 24 hours under anaerobic conditions at 37°C. Subsequently, transformation reaction mixtures were plated on BHI-agar containing appropriate antibiotics (kanamycin: 500 µg/mL or erythromycin: 10 µg/mL) for selection. Once the plates had dried, the cells were overlaid with a thin layer of agar by adding 1 mL of cooled BHI-agar with antibiotics. The plates were incubated at 37°C under anaerobic conditions to select transformants for 4–5 days.

For genotyping, genomic DNA was isolated from 2 mL of saturated cultures. In brief, cells were precipitated by centrifugation at 10,000 rpm for 10 minutes at room temperature. They were then resuspended in 200 µL of resuspension buffer (20 mM EDTA, 200 mM Tris-HCl, and 2% Triton-X 100) and lysed with 200 µL of AL lysis buffer (Qiagen, 19075). DNA was precipitated by adding 1 mL of 100% ethanol supplemented with 100 mM of sodium acetate. Following washing and drying, the DNA was dissolved in 100 µL of water and used for PCR and/or whole-genome sequencing.

To generate deletion mutants Δ*f1fo* in Δ*trkA1-H1* and Δ*trkA2-H2*, the Δ*trkA1-H1* and Δ*trkA2-H2* mutants were first generated using *Erm*^r^. After confirmation of deletion of Δ*trkA1-H1* and Δ*trkA2-H2*, Δ*f1fo* of *kan* resistance construct was subsequently transformed into Δ*trkA1-H1* or Δ*trkA2-H2*. To generate deletion mutants of both *trkA1-H1* and *trkA2-H2*, *trkA1*, *trkH1*, or Δ*trkA1-H1* of erythromycin resistance, constructs were transformed into a Δ*trkH2* mutant of kan resistance background (1). To isolate Δ*f1fo* and Δ*trkA1-H1* mutants in the SK405 and SK1058 strains, we employed the same PCR construct used for deleting *f1fo* and *trkA1-H1* in SK36, as there is >90% identity at the flanking regions between SK36, SK1058, and SK405.

### Strain passage

Antibiotic-resistant colonies from selection plates were transferred to 1 mL of BHI containing antibiotics and grown for 2–8 days under anaerobic conditions at 37°C. The duration depended on when the OD_600_ reached 0.1–0.5, which was referred to as P0. A 300 µL aliquot of the cell culture was then transferred to 3 mL of BHI containing antibiotics, and growth continued until saturation, resulting in P1. P1 was mixed by pipetting five times with a P-1000 pipette, and 50 µL of the mixed P1 was transferred to 1 mL of BHI containing antibiotics. Growth proceeded until the cell culture reached an OD_600_ of 0.1–0.5, producing P2. The latter process was repeated sequentially from P3 to P6, where the volume of P2 to P5 was 1 mL, and the final passage, P6, was 3 mL. Cell cultures of P1 and P6 were stored at −80°C in glycerol at the final concentration of 20%. DNA extractions and genome sequencing were conducted in P6 and/or P1 when indicated.

For passages involving WT, glycerol stocks from −80°C were transferred to 1 mL of BHI and grown for 24 hours, resulting in T0 (see Fig. S5a). A 50 µL aliquot of T0 cell culture was aliquoted into six 1.5 mL tubes, each containing 1 mL of BHI, and grown for 24 hours, resulting in T1. A 50 µL aliquot of T1 cell culture was inoculated into 1 mL of BHI and grown for 24 hours, leading to T2. This process was repeated for T3–T9.

### Whole-genome sequencing and variant calling

Whole-genome sequencing was carried out by the Genome Core at Virginia Commonwealth University or by SeqCenter (https://www.seqcenter.com/) using the shotgun method with 2 × 150 paired-end sequencing. Whole-genome sequences with an average coverage of ≥100 were used for subsequent analysis.

Fastq files were aligned to an updated SK36 reference genome sequence (accession number GCA_050226025.1) using Geneious Prime software (https://www.geneious.com/) after trimming via the BBDuk method. Variations in the genome were exported from Geneious Prime. To identify the mutated segment, the frequencies (percentages) of all mutations belonging to a certain segment were assessed, and the greatest value of percentages was assigned to that segment. For the deletions, the read coverages were calculated by averaging the base coverages that were 100 bp from the flanking sequence.

### RT-qPCR

Three milliliter of late-log phase bacterial culture (OD₆₀₀ ≈ 0.4), grown in BHI under anaerobic conditions at 37°C, was mixed with 6 mL of RNAprotect Tissue Reagent (Qiagen, Cat. No. 76104) and harvested by centrifugation at 1,000 × *g* for 10 minutes. Total RNA was extracted using the RNeasy Mini Kit (Qiagen, Cat. No. 74104) according to the manufacturer’s instructions. For cDNA synthesis, 0.5 µg of total RNA was reverse-transcribed using the High-Capacity cDNA Reverse Transcription Kit (Applied Biosystems, Cat. No. 4374966) in a 20 µL reaction. Quantitative PCR was performed using 5 µL of 1:20 diluted cDNA and SYBR Green master mix (Quantabio, Cat. No. 95071-012) in a 20 µL reaction on an Applied Biosystems 7500 Real-Time PCR System. Relative expression levels of *acpS* were calculated using the ΔΔCT method, with *gyrA* serving as the endogenous reference gene. Results were normalized to the expression levels observed in the wild-type-like strain Δ*SSA_0169*.

### Glucose and fructose assay for pfkA

The wild-type strain (WT, SSA_0169 mutant-like) was inoculated from glycerol stock and grown overnight in BHI supplemented with 500 µg/mL kanamycin under anaerobic conditions at 37°C. The *pfkA* mutant, which exhibited extremely slow growth, was inoculated from glycerol stock and cultured for one day in 1 mL BHI containing 500 µg/mL kanamycin under the same conditions. Cells were condensed by centrifugation at 4,000 rpm for 2 min, after which 900 µL of supernatant was removed. After mixing by pipetting with 1 mL tip for three times, 10 µL of cells were transferred to 990 µL TY medium (3% [wt/vol] tryptone and 0.5% [wt/vol] yeast extract) to generate a 100-fold dilution, which was used as the inoculum. CFU counts of inocula were determined by serial dilution and plating on agar plates.

For growth assays, 10 µL of inoculum was added to 990 µL TY medium supplemented with either 20 mM glucose, 20 mM fructose, or no sugar. Cultures were incubated anaerobically at 37°C for 24 h, and CFU counts were determined by serial dilution and plating.

### Statistics

For single pairwise comparisons, two-tailed Welch’s *t*-tests were used to detect significant differences (*P* < 0.05). For analyses involving multiple groups, one-way (single variable) or two-way (two variables) ANOVA followed by Tukey’s multiple comparisons test was applied (*P* < 0.05). Statistically significant differences between groups are indicated by the letters “a,” “b,” “c,” and “d,” following the compact letter display method in GraphPad Prism (https://www.graphpad.com/guides/prism/latest/user-guide/compact_letter_display.htm).

## Data Availability

All data needed to evaluate the conclusions in the paper are present in the paper and/or the supplemental files. The genome sequence data were deposited to GenBank (PRJNA1146876). All the transgenic materials, including the essential gene deletion mutants, are available from the authors upon request.

## SUPPLEMENTAL MATERIAL

Supplemental Figures mbio.03005-25-s0001.pdf Figures S1 to S10.

Supplemental Text mbio.03005-25-s0002.docx Supplemental results and discussion.

Supplemental Tables mbio.03005-25-s0003.xlsx Tables S1 to S13.

ASM does not own the copyrights to Supplemental Material that may be linked to, or accessed through, an article. The authors have granted ASM a non-exclusive, world-wide license to publish the Supplemental Material files. Please contact the corresponding author directly for reuse.
