# Supplementary material for: Experimental evolution of gene essentiality in bacteria
Source: mBio. 2025 Oct 31;16(12):e03005-25. doi: 10.1128/mbio.03005-25 (PMC12691585; doi:10.1128/mbio.03005-25)
Supplement: Supplemental Figures — Figures S1 to S10. [file mbio.03005-25-s0001.pdf]

Fig.S1

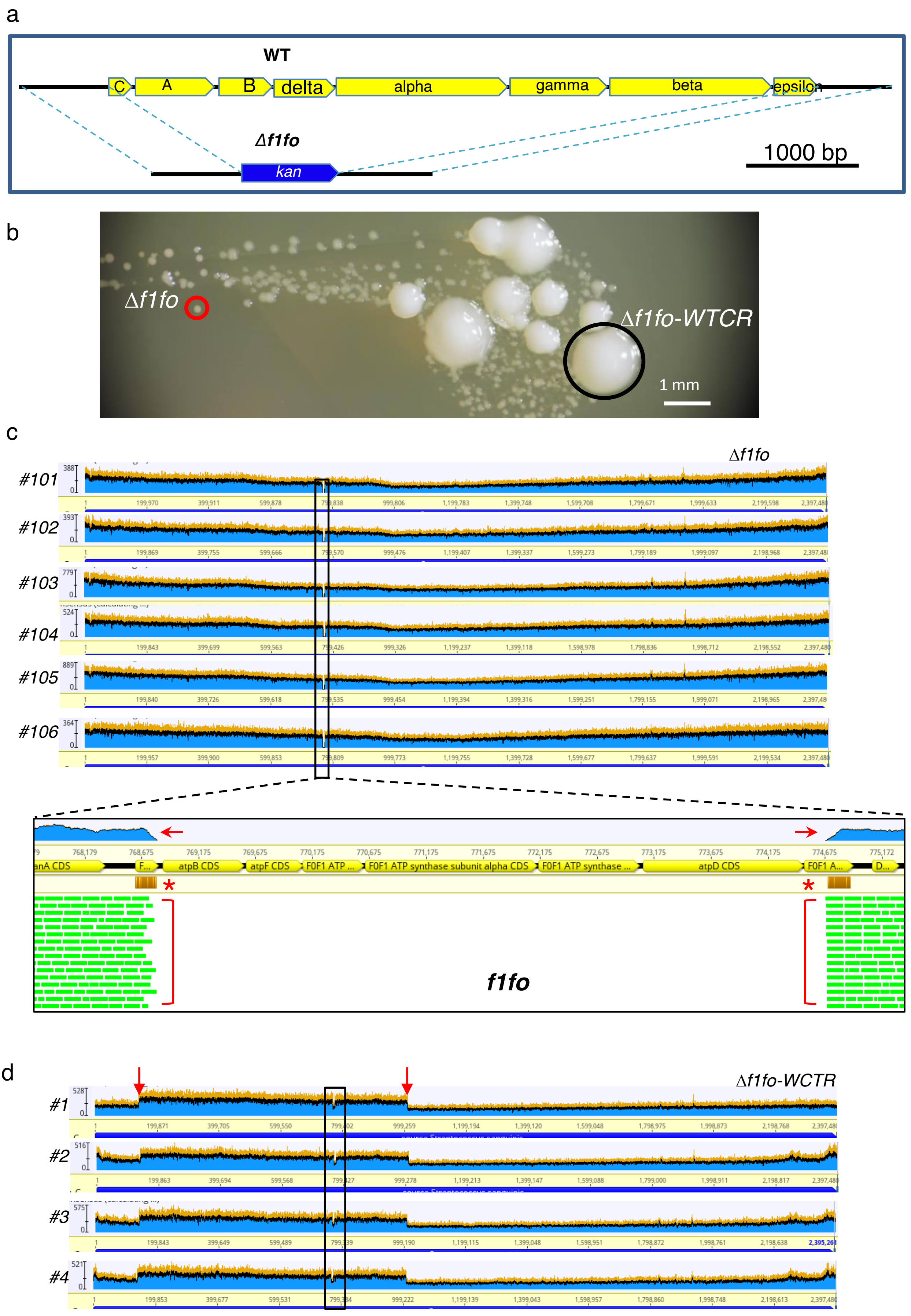
a
WT
C
A
B
delta
alpha
gamma
beta
epsilon
Δf1fo
kan
1000 bp
b
Δf1fo
Δf1fo-WTCR
1 mm
c
Δf1fo
#101
#102
#103
#104
#105
#106
anA CDS
F...
atpB CDS
atpF CDS
F0F1 ATP ...
F0F1 ATP synthase subunit alpha CDS
F0F1 ATP synthase ...
atpD CDS
F0F1 A...
D...
f1fo
d
Δf1fo-WCTR
#1
#2
#3
#4

Supplemental figures and legends.

Supplementary Fig.1. Genotyping and complementation of mutants deleted of *f1fo*.

(a) Strategy to delete the entire eight-gene *f1fo* operon by homologous recombination. The yellow blocks indicate the eight F1Fo subunit genes in WT (upper). The blue block indicates the *kan* gene in the Δ*f1fo* mutants (lower).

(b) Two types of colonies, small and large, as demonstrated for *f1fo* deletion, appear on selection plates after 5 days of growth. Only the small colonies represent true deletions, denoted as Δ*f1fo* (red circle). The large colonies are "double-band mutants," containing both the replacement of the original *f1fo* with a *kan* gene and a wild-type *f1fo* copy, or they are mutants that did not contain a *kan* gene, but contained point mutations in other genes, denoted as Δ*f1fo-WTCR* (black circle, 'WTCR' for wild-type copy retained).

(c) Whole-genome sequencing to confirm the genotypes of the small colonies of the Δ*f1fo* mutants. Black box indicates the location of deleted *f1fo* region. Enlarged image showing alignment of sequencing reads to the reference genome of *S. sanguinis* SK36. The blue graphic above the sequence coordinates (indicated by a red arrow) indicates the extent of sequence-read coverage; the vertical gold bars indicate the mismatches (indicated by a star), and the bracket indicates the aligned sequence reads.

(d) Whole-genome sequencing of the large, Δ*f1fo*-DB colonies. Black box indicates the location of *f1fo* region. Red arrows indicate the two directly repeated *ugpC* genes, encoding glycerol-3phosphate ABC transporter.

Fig.S2

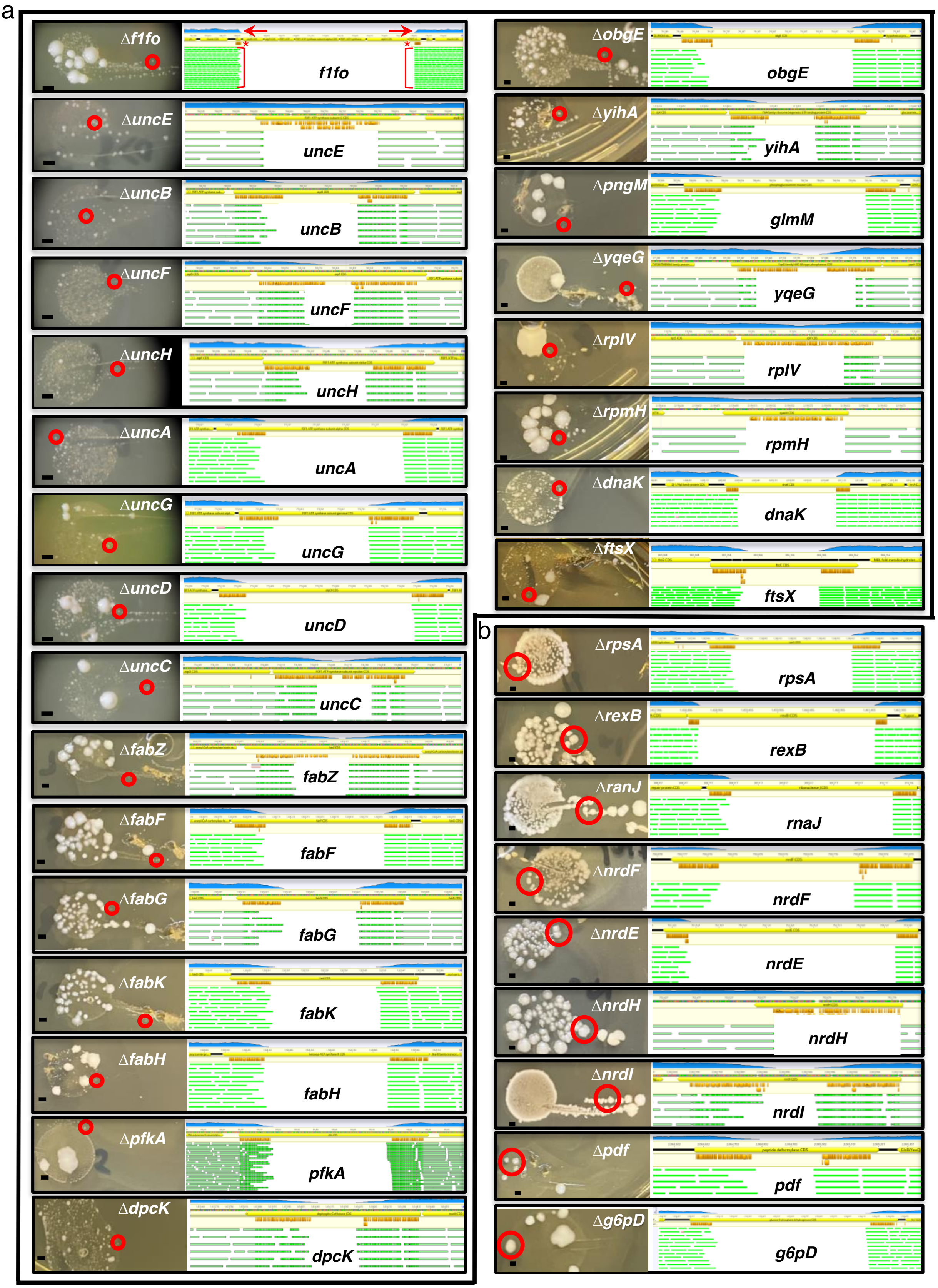
a
Δf1fo
f1fo
ΔuncE
uncE
ΔuncB
uncB
ΔuncF
uncF
ΔuncH
uncH
ΔuncA
uncA
ΔuncG
uncG
ΔuncD
uncD
ΔuncC
uncC
ΔfabZ
fabZ
ΔfabF
fabF
ΔfabG
fabG
ΔfabK
fabK
ΔfabH
fabH
ΔpfkA
pfkA
ΔdpcK
dpcK
ΔobgE
obgE
ΔyihA
yihA
ΔpngM
glmM
ΔyqeG
yqeG
ΔrplV
rplV
ΔrpmH
rpmH
ΔdnaK
dnaK
ΔftsX
ftsX
b
ΔrpsA
rpsA
ΔrexB
rexB
ΔranJ
rnaJ
ΔnrdF
nrdF
ΔnrdE
nrdE
ΔnrdH
nrdH
ΔnrdI
nrdI
Δpdf
pdf
Δg6pD
g6pD

Supplementary Fig.2. Isolation of viable mutants deleted for essential genes and genotyping by whole-genome sequencing.

(a-b) Colonies of transformants (left side of each panel) and whole-genome sequencing of colonies (right side of each panel). Red circles (left) indicate the colonies picked for whole genome sequencing. For genome sequencing (right), the blue graphic above the sequence coordinates (indicated by a red arrow in the top panel of a) indicates the extent of coverage, the gold bars indicate the mismatches, and the bracket indicates the aligned sequence reads. Bacterial colonies on selective agar plate for five days. (a) Gene deletion mutants with severe growth defects. (b) Gene deletion mutants with robust growth. Scale bar: 1 mm. The image of Δ*f1fo* transformation is a duplicate of **Fig. 1b** to demonstrate the transformants in selection medium; Δ*f1fo* alignment is a duplication of **Fig.S1c**.

Fig.S3

a b

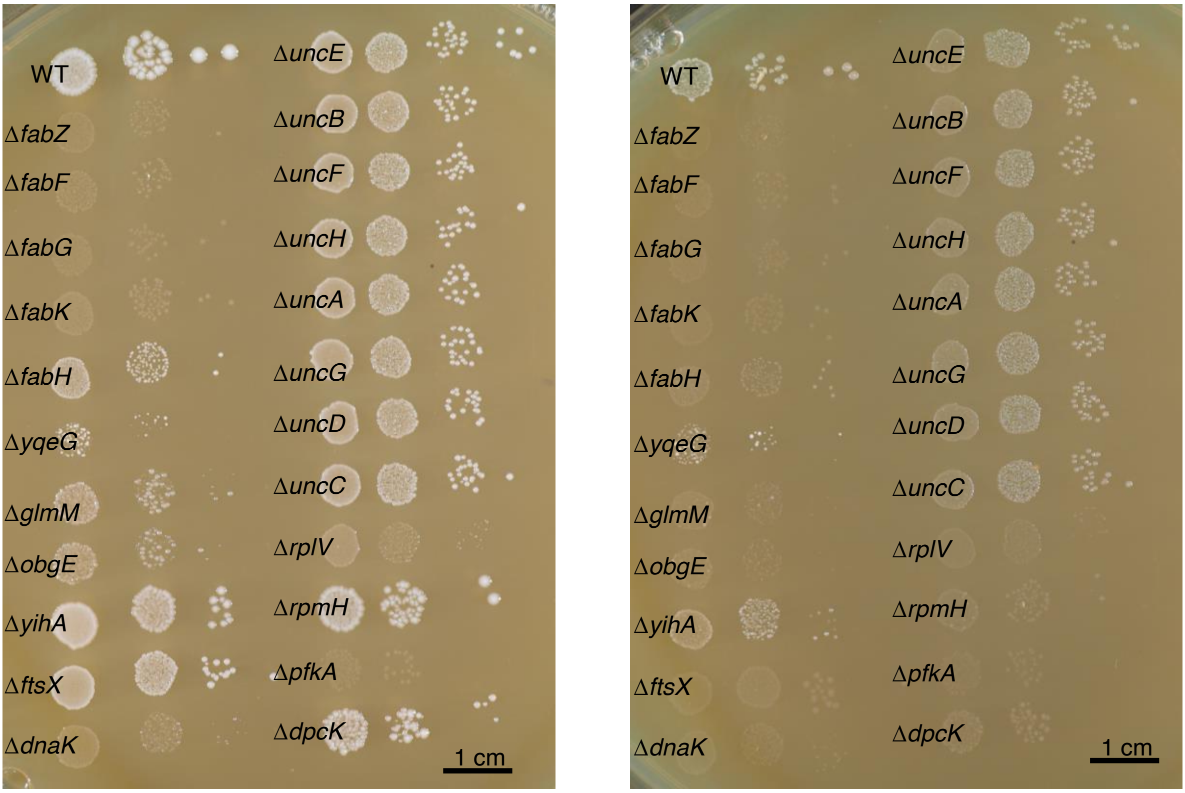
WT
ΔfabZ
ΔfabF
ΔfabG
ΔfabK
ΔfabH
ΔyqeG
ΔglmM
ΔobgE
ΔyihA
ΔftsX
ΔdnaK
ΔuncE
ΔuncB
ΔuncF
ΔuncH
ΔuncA
ΔuncG
ΔuncD
ΔuncC
ΔrplV
ΔrpmH
ΔpfkA
ΔdpcK
1 cm
WT
ΔfabZ
ΔfabF
ΔfabG
ΔfabK
ΔfabH
ΔyqeG
ΔglmM
ΔobgE
ΔyihA
ΔftsX
ΔdnaK
ΔuncE
ΔuncB
ΔuncF
ΔuncH
ΔuncA
ΔuncG
ΔuncD
ΔuncC
ΔrplV
ΔrpmH
ΔpfkA
ΔdpcK
1 cm

Supplementary Fig. 3. Effect of oxygen on the growth of WT and mutants deleted of essential genes.

(a-b) Growth of WT and mutants deleted of 23 essential genes under anaerobic (a) and microaerobic (b) conditions. 0.1 OD_{600} of cells in a volume of 2 µl were spotted directly (first column) or diluted 20-fold (column 2), or 400-fold (column 3) and grown on BHI-agar for two days. Scale bar is 1 cm.

Fig.S4

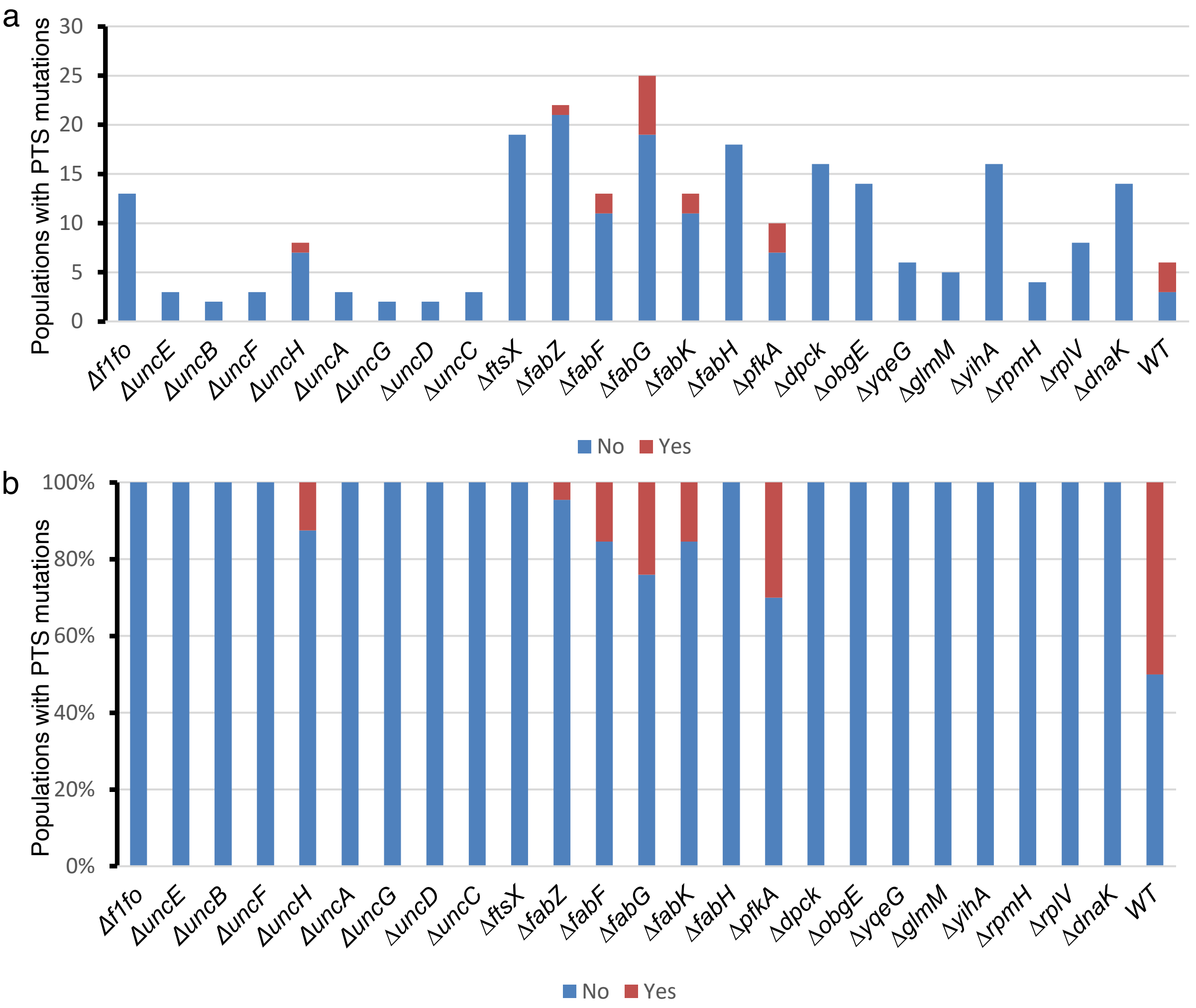
a
Populations with PTS mutations
0
5
10
15
20
25
30
Δf1fo
ΔuncE
ΔuncB
ΔuncF
ΔuncH
ΔuncA
ΔuncG
ΔuncD
ΔuncC
ΔftsX
ΔfabZ
ΔfabF
ΔfabG
ΔfabK
ΔfabH
ΔpfkA
Δdpck
ΔobgE
ΔyqeG
ΔglmM
ΔyihA
ΔrpmH
ΔrplV
ΔdnaK
WT
No
Yes
b
Populations with PTS mutations
0%
20%
40%
60%
80%
100%
Δf1fo
ΔuncE
ΔuncB
ΔuncF
ΔuncH
ΔuncA
ΔuncG
ΔuncD
ΔuncC
ΔftsX
ΔfabZ
ΔfabF
ΔfabG
ΔfabK
ΔfabH
ΔpfkA
Δdpck
ΔobgE
ΔyqeG
ΔglmM
ΔyihA
ΔrpmH
ΔrplV
ΔdnaK
WT
No
Yes

Supplementary Fig.4. Populations with and without mutations in three PTS mannose/fructose/sorbose transporter subunits.

Number (a) and percentage (b) of populations of WT and essential-gene mutants that acquired mutations in any of three PTS mannose/fructose/sorbose transporter subunits, namely SSA_1918, SSA_1919, and SSA_1920.

Fig.S5

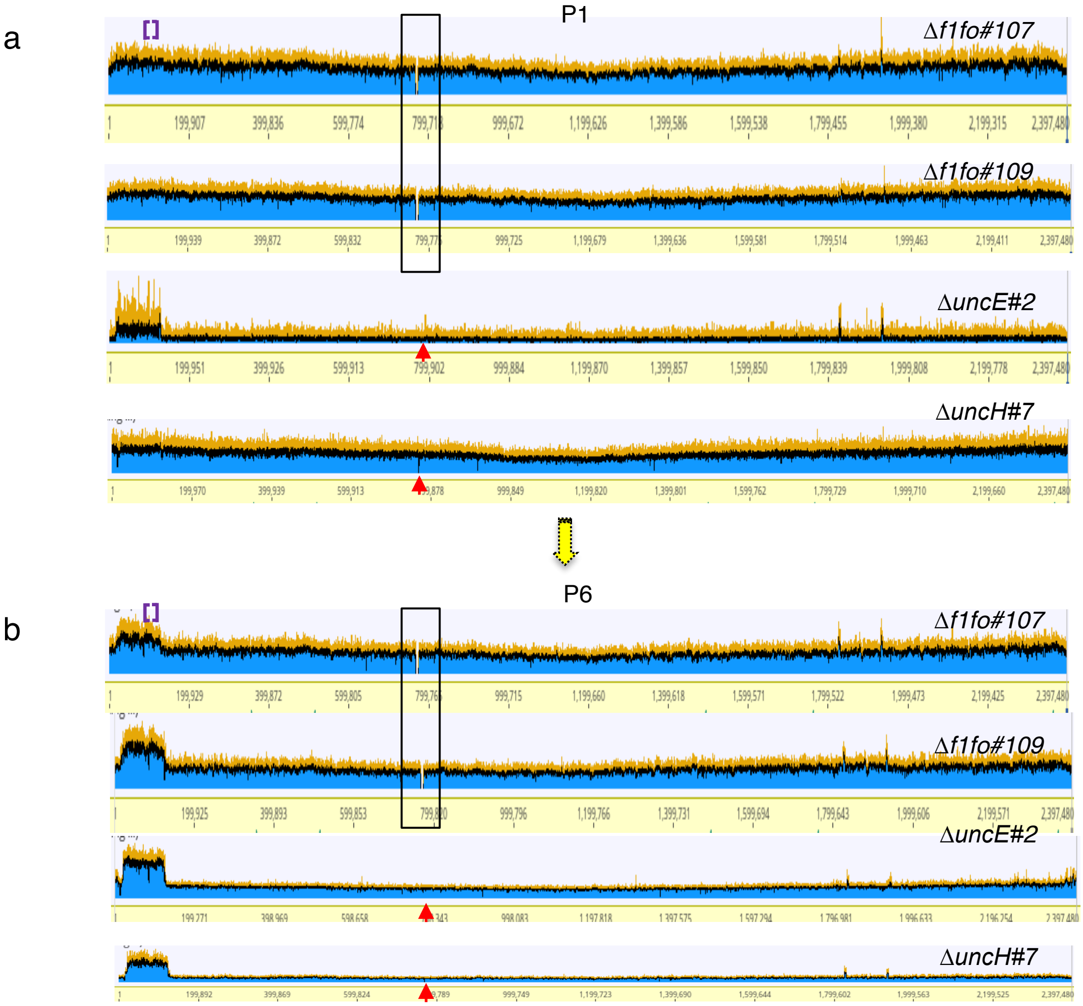
a
P1
Δf1fo#107
Δf1fo#109
ΔuncE#2
ΔuncH#7
b
P6
Δf1fo#107
Δf1fo#109
ΔuncE#2
ΔuncH#7

Supplementary Fig.5. Appearance of *v1vo* region gene duplications during passage. (Related to Figure 3a)

(a-b) Alignment of sequence reads in populations of *Δf1fo#107, Δf1fo#109, uncE#2* and *uncH#7* at P1 (a) and P6 (b). The black box indicates the deleted *f1fo* region and the red arrows indicate the deleted *uncE* or *uncH* gene. Purple brackets indicate the *v1vo* region.

Fig.S6

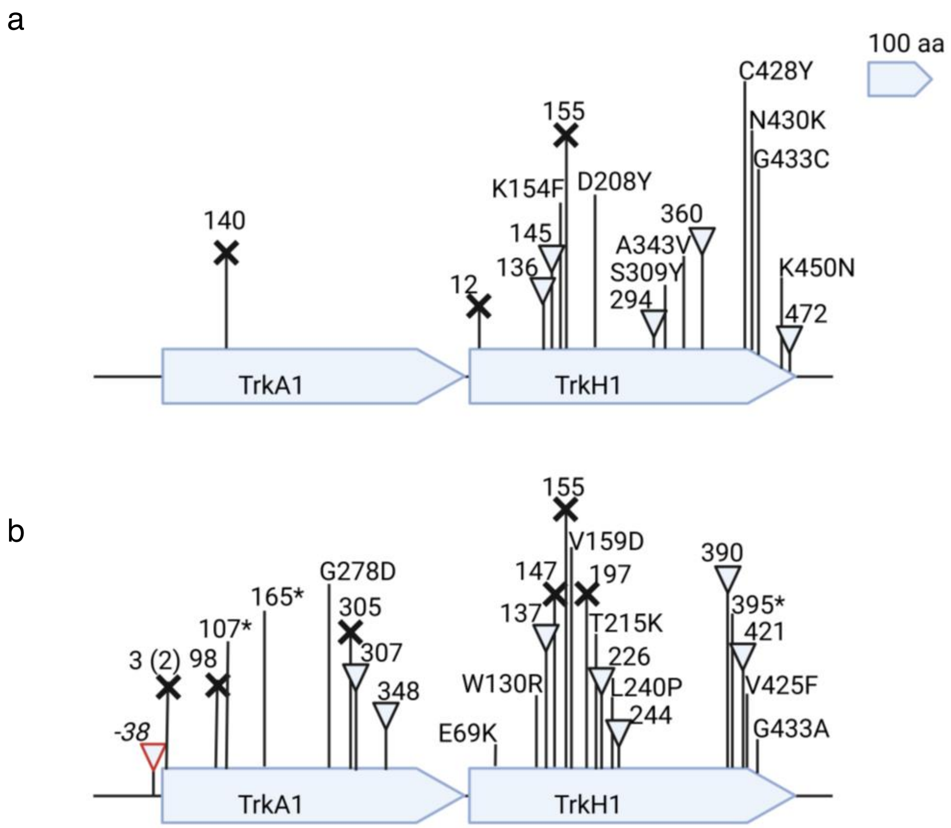
a
100 aa
140
12
136
145
K154F
155
D208Y
294
S309Y
A343V
360
C428Y
N430K
G433C
K450N
472
TrkA1
TrkH1
b
-38
3 (2)
98
107*
165*
G278D
305
307
348
E69K
W130R
137
147
155
V159D
197
T215K
226
L240P
244
390
395*
421
V425F
G433A
TrkA1
TrkH1

Supplementary Fig.6. Mutations in the *trkA1-H1* region.

a-b, Mutations in the *trkA1-H1* region found in evolved populations of *Δf1fo* (**a**) or F1Fo subunit deletion mutants **(b).** Vertical bars represent mutation positions at the amino acid number indicated. Amino acid substitutions are denoted by two letters with a number in between. Numbers in parentheses indicate the number of identical mutations obtained from independent populations. Truncations (i.e., nonsense mutations) are marked by an asterisk (*). Insertions are represented by inverted triangles above the bars, while deletions are indicated by crosses. Note that all insertions or deletions resulted in frameshift mutations, except for one at position 136 of TrkH1 for one *Δf1fo* population (**a**), which caused an in-frame insertion of a single amino acid, and a second one located 38 bp upstream of *trkA1-H1* in one F1Fo subunit deletion population (**b**).

Fig.S7

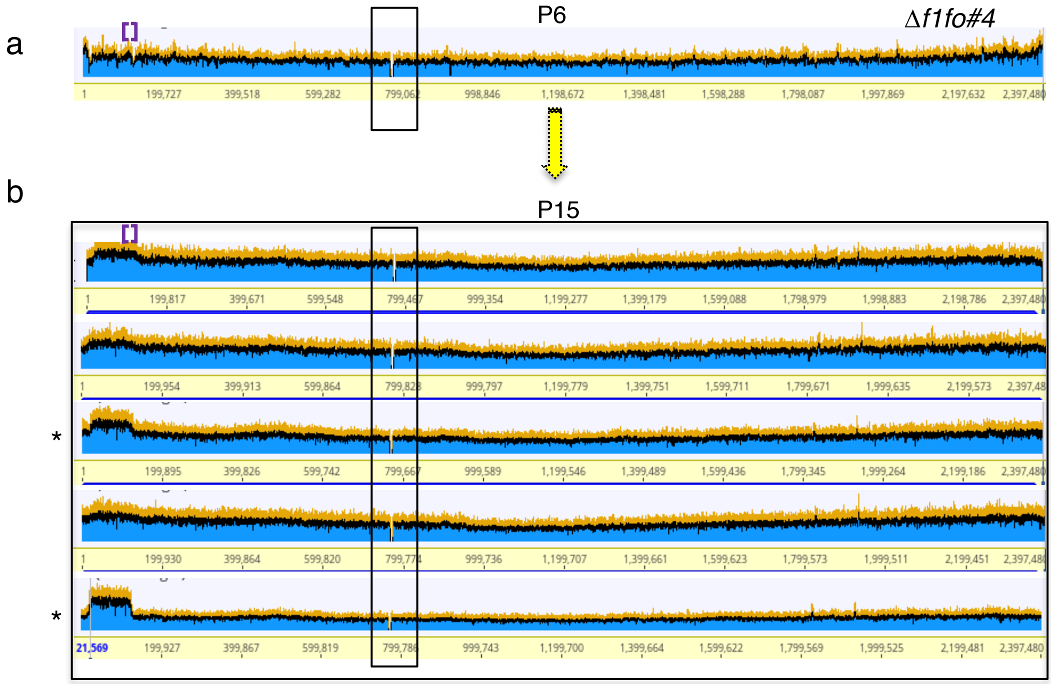
a
P6
Δf1fo#4
1
199,727
399,518
599,282
998,846
1,198,672
1,398,481
1,598,288
1,798,087
1,997,869
2,197,632
2,397,480
b
P15
1
199,817
399,671
599,548
999,354
1,199,277
1,399,179
1,599,088
1,798,979
1,998,883
2,198,786
2,397,480
199,954
399,913
599,864
999,797
1,199,779
1,399,751
1,599,711
1,799,671
1,999,635
2,199,573
*
199,895
399,826
599,742
999,589
1,199,546
1,399,489
1,599,436
1,799,345
1,999,264
2,199,186
199,930
399,864
599,820
999,736
1,199,707
1,399,661
1,599,623
1,799,573
1,999,511
2,199,451
21,569
199,927
399,867
599,819
999,743
1,199,700
1,399,664
1,599,622
1,799,569
1,999,525
2,199,481

Supplementary Fig.7. Appearance of *v1vo* region gene duplications during passage. (Related to Figure 3b)

(a-b) Alignment of sequence reads of the P6 population in *Δf1fo#4* (a) and five derived populations from P15 (b). Black rectangles represent the deleted *f1fo* region. Asterisks (*) indicate the two populations with *v1vo* region gene duplications. Purple brackets indicate the *v1vo* region.

Fig.S8

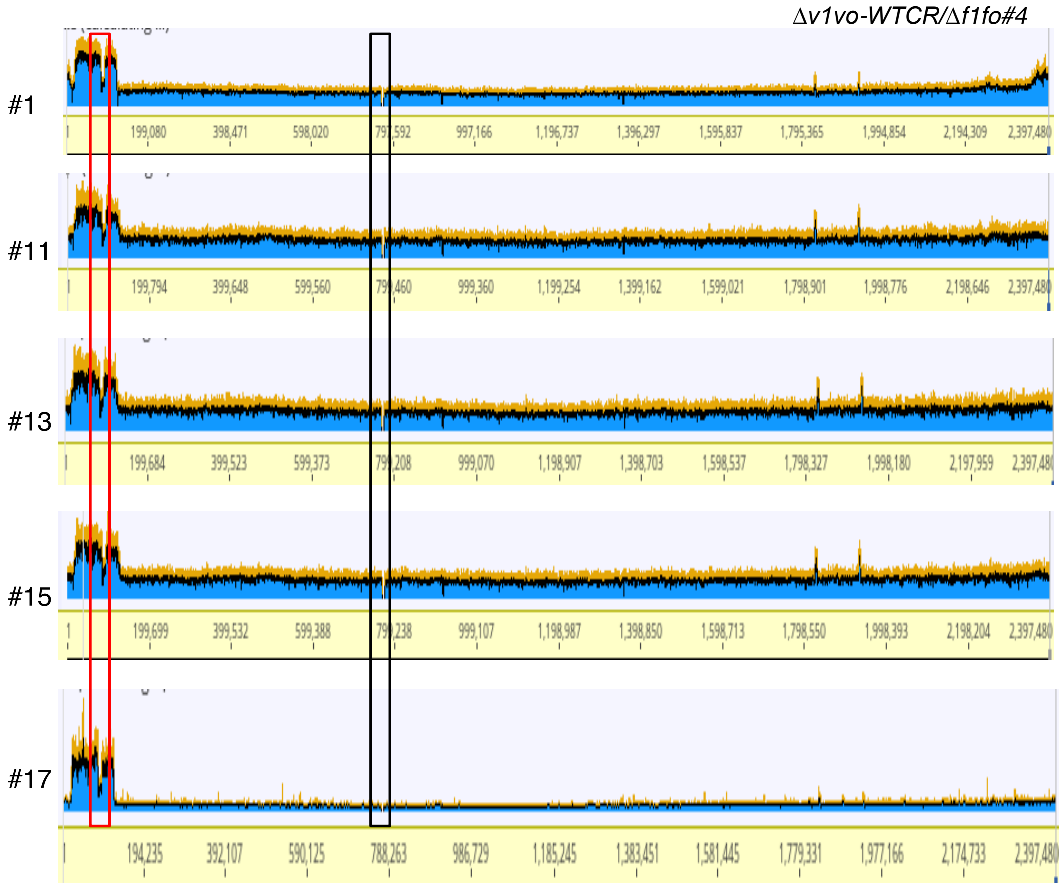

Supplementary Fig.8. Gene duplication in *Δv1vo-DB/Δf1fo#4* populations (related to Figure 3c).

Alignment of sequence reads of five additional *Δv1vo-WTCR/Δf1fo#4* populations. The black box indicates the deleted *f1fo*. Purple brackets indicate the gene duplication of *v1vo* region when one copy of *v1vo* was intentionally deleted from ***Δf1fo#4***.

Fig. S9

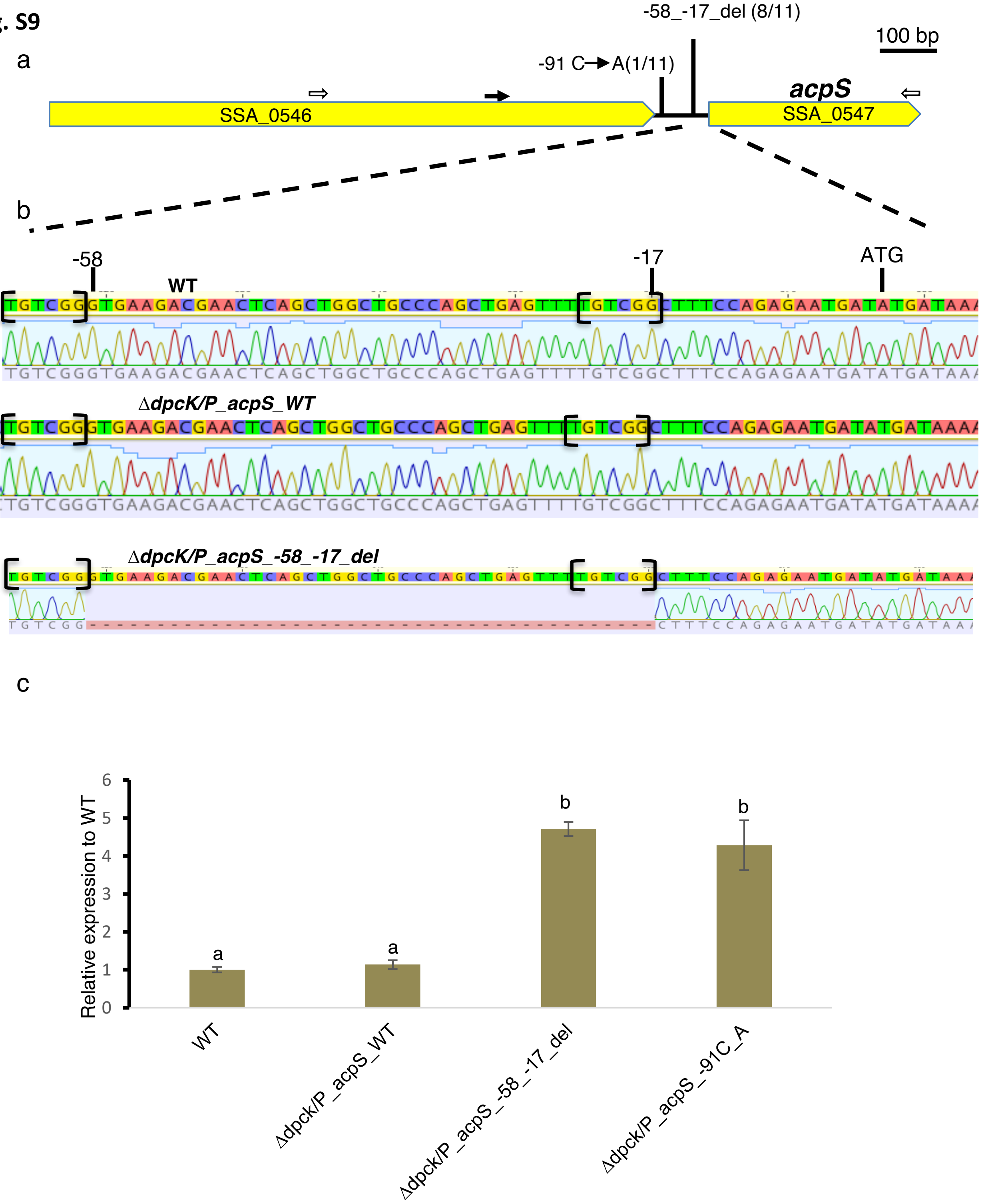

a
-58_-17_del (8/11)
100 bp
-91 C→A(1/11)
acpS
SSA_0546
SSA_0547
b
-58
-17
ATG
WT
TGTCGGGTGAAGACGAACTCAGCTGGCTGCCCAGCTGAGTTTTGTCGGCTTTCCAGAGAATGATATGATAAA
ΔdpcK/P_acpS_WT
TGTCGGGTGAAGACGAACTCAGCTGGCTGCCCAGCTGAGTTTTGTCGGCTTTCCAGAGAATGATATGATAAAA
ΔdpcK/P_acpS_-58_-17_del
TGTCGG
CTTTCCAGAGAATGATATGATAA
c
Relative expression to WT
0
1
2
3
4
5
6
a
a
b
b
WT
Δdpck/P _acpS_WT
Δdpck/P _acpS _-58 _-17 _del
Δdpck/P _acpS _-91C_A

Supplementary Fig.9. Location and effects of upstream *acpS* mutations in *ΔdpcK* mutants

(a) Strategy for sequencing the 106 bp upstream intergenic region of *acpS*. Yellow blocks represent the upstream ORF and the *acpS* gene. Bold unfilled arrows indicate the locations of primers used for PCR, while bold filled arrows indicate the locations of primers used for sequencing. Two types of mutations were identified: a deletion spanning −58 to −17 relative to *acpS* in eight evolved populations, and a C-to-A substitution at position −91 in one population. The numbers in parentheses indicate the frequency of each mutation among the total *ΔdpcK* mutant populations.

(b) Alignment of DNA sequences from the WT strain and two Δ*dpcK* mutants, one of which carries a −58 to −17 upstream deletion of *acpS* (lower). Black brackets mark the 6-bp direct repeats (TGTCGG), and the dashed line indicates the −58 to −17 deletion upstream of the *acpS* start codon in the Δ*dpcK*.

(c) Relative expression of *acpS* in the WT strain and three representative ΔdpcK mutants: one carrying a −58 to −17 upstream deletion of *acpS*, one carrying a −91 C-to-A substitution, and one without detectable upstream mutations. Data represent the mean of three biological replicates, each with three technical repeats. Error bars indicate standard deviations. Different letters indicate statistically significant differences ($P \leq 0.05$), determined by one-way ANOVA followed by Tukey’s multiple comparisons test. Plots that do not share a letter (a or b) represent groups that are significantly different.

Fig. S10

a

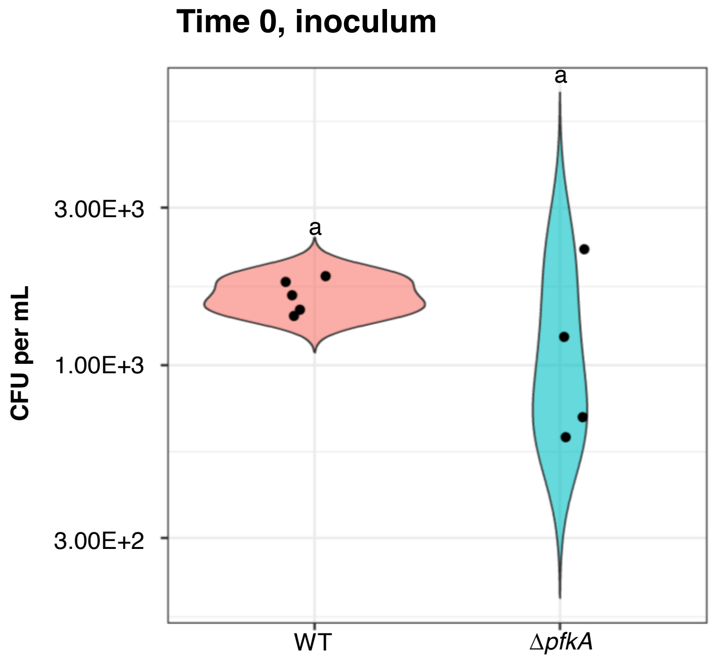
Time 0, inoculum
CFU per mL
3.00E+3
1.00E+3
3.00E+2
a
a
WT
ΔpfkA

b

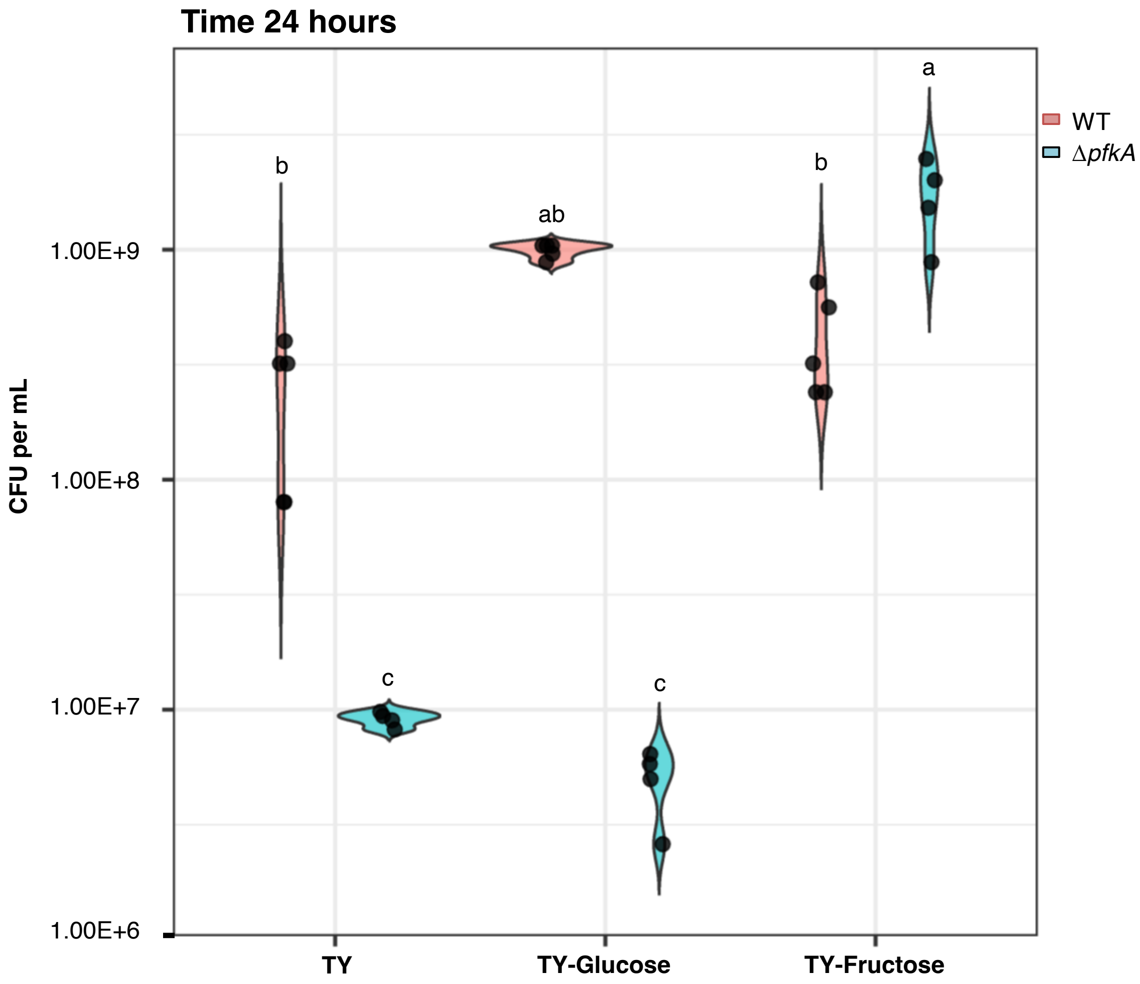
Time 24 hours
CFU per mL
1.00E+9
1.00E+8
1.00E+7
1.00E+6
a
b
ab
b
c
c
WT
ΔpfkA
TY
TY-Glucose
TY-Fructose

Supplementary Fig.10. Rescue of *ΔpfkA* mutants by fructose

(a) Violin plots of CFU counts of the inocula for WT and *ΔpfkA*. Inocula were prepared by diluting 10 µL of culture into 990 µL of TY medium. Statistical significance ($P < 0.05$) was assessed by two-tailed Welch's t-tests. Violin plots that do share a same letter (a) represent groups that are not significantly different.

(b) Violin plots of CFU counts for WT and *ΔpfkA* after 24 h of anaerobic growth at 37 °C in TY medium supplemented with 20 mM glucose, 20 mM fructose, or no addition. Different letters indicate statistically significant differences ($P \leq 0.05$), determined by two-way ANOVA followed by Tukey's multiple comparisons test. Violin plots that do not share a letter (a, b, or c) represent groups that are significantly different.
