# Supplementary material for: Experimental evolution of gene essentiality in bacteria
Source: mBio. 2025 Oct 31;16(12):e03005-25. doi: 10.1128/mbio.03005-25 (PMC12691585; doi:10.1128/mbio.03005-25)
Supplement: Supplemental Text — Supplemental results and discussion. [file mbio.03005-25-s0002.docx]

**Supplemental Results and Discussion**

**Authors**:

Liang Bao^1, *^, Zan Zhu^1^, Ahmed Ismail^1^, Bin Zhu^2^, Vysakh Anandan^1^, Marvin Whiteley^3^, Todd Kitten^1^, Ping Xu^1, *^

**Possible explanations for mutations observed in additional slow-growing essential gene mutants**

Given the potential role of parallel evolution for fitness improvement,(1-3) we next sought to identify the most commonly mutated segments for each slow growing essential gene mutant (Supplemental Table 7) and, when possible, to propose mechanisms by which these mutated segments improved growth.

Potential suppressor mutations in *∆fab operon mutants*

The *fabH, fabF, fabG, fabZ, and fabK* genes belong to a FASII operon containing twelve ORFs in total, with a MarR family transcriptional regulator gene (*fabT*) upstream and seven other genes. In the evolved populations of *∆fabZ, ∆fabF, ∆fabG, ∆fabK,* and *∆fabH*, we observed a significant enrichment of mutations in the other genes in this operon that encode additional components of the FASII system (Supplemental Table 2 and 7). This result suggests that when one component of the FASII system is deleted, this can trigger genome changes in other FASII components, leading to fitness improvement.

There is a notable number of populations containing a duplication of approximately 103 kb (coordinate: 22,738-125,834) in the *∆fabH, ∆fabF,* and *∆fabG* mutants, which includes one ORF encoding an acyltransferase and one ORF encoding acyl carrier protein (Fig. 2b). Additionally, a duplicated region of 10.1 kb (coordinate: 1,093,824-1,103,958) is shared by *∆fabZ #1* and *∆fabZ #3* and contains acyl-acyl carrier protein thioesterase (Fig. 2b).

Potential suppressor mutations in *∆obgE suppressors*

ObgE belongs to the TRAFAC (Translation Factor Association) class of P-loop GTPases, which is highly conserved and essential in most bacteria. (4) The ObgE GTPase is known to possess a wide range of functions, with some of the most significant roles including sporulation initiation in *Bacillus subtilis*, (5) aerial mycelium formation in *Streptomyces*, (6, 7) bacterial persistence in *E. coli* and *Pseudomonas aeruginosa*, (8) involvement in ribosome biogenesis, (9, 10) participation in DNA replication, (11-13) and contributions to chromosome segregation and cell division. (14)

Among the fourteen evolved *∆obgE* populations, eight populations had substitution mutations upstream of *rpoB* (seven were an A to T mutation 321 bp upstream of the start codon, and the other was an A to G mutation 24 bp upstream), and two populations contained substitution mutations within the *rpoB* ORF (one at 905 bp and the other at 1058 bp from the start of the 3567 bp ORF). In total, 71.4% of the populations had mutations within or upstream of *rpoB* (Supplemental Table 2 and 7). The *rpoB* gene codes for the beta-subunit of RNA polymerase I. In one of these populations, *∆obgE#5*, a 37 kb gene duplication was observed (coordinate: 1,775,394-1,812,805), encompassing thirty ORFs, including one annotated as Der GTPase (Fig. 2b). It was reported that overexpression of *der* or *obgE* can suppress growth defects of a mutant lacking *rrmJ*, which encodes the methyltransferase for U2552 of the 23S rRNA. (15) From this, we hypothesize that the duplication may partially compensate for the loss of ObgE function by increasing *der* copy numbers, and a reduction in the rate of RNA synthesis is a significant fitness adaptation when ObgE is defective.

Potential suppressor mutations in *ΔftsX suppressors*

FtsEX is crucial in regulating divisome assembly and activation at the plasma membrane and cell wall. (16) In the evolved *ΔftsX* populations, mutations in the *ftsE* gene of the *ftsEX* operon and the *vicRK (vicK/walK* and *vicR/walR)* two-component system (TCS) frequently occurred. Mutations in *ftsE* were present in eight out of nineteen evolved *ΔftsX* populations (42.1%); in one of these populations, *ΔftsX#13*, a deletion mutation at 51 bp of the 693 bp *ftsE* ORF leads to a frameshift in the N-terminal region. This observation suggests that in the absence of *ftsX*, *ftsE* does not function properly and is deleterious to the cell. Although *vicR* and *vicK* compose a TCS, *vicK* is not essential, while *vicR* is essential. (17, 18) Fifteen populations contained *vicK* mutations (with one frameshift and one truncation), and one population contained a *vicR* mutation (an amino acid substitution, M54V), collectively representing 89.5% of the evolved populations (Supplemental Table 2 and 7). It was reported that FtsEX interacts with the cell wall component peptidoglycan hydrolase, PcsB, which is positively regulated by the *vicRK/walRK* two-component regulatory system. (19) Therefore, we hypothesize that when FtsEX is absent and PcsB cannot function properly, there is a need to inhibit the activity of PcsB's upstream regulatory factor, VicRK.

**References:**

1. Chevin, L.M., Martin, G., and Lenormand, T. (2010). Fisher's model and the genomics of adaptation: restricted pleiotropy, heterogenous mutation, and parallel evolution. Evolution *64*, 3213-3231.

2. Wichman, H.A., Badgett, M.R., Scott, L.A., Boulianne, C.M., and Bull, J.J. (1999). Different trajectories of parallel evolution during viral adaptation. Science *285*, 422-424.

3. Stern, D.L. (2013). The genetic causes of convergent evolution. Nat Rev Genet *14*, 751-764.

4. Deckers, B., Vercauteren, S., De Kock, V., Martin, C., Lazar, T., Herpels, P., Dewachter, L., Verstraeten, N., Peeters, E., Ballet, S., et al. (2023). YbiB: a novel interactor of the GTPase ObgE. Nucleic Acids Res *51*, 3420-3435.

5. Vidwans, S.J., Ireton, K., and Grossman, A.D. (1995). Possible role for the essential GTP-binding protein Obg in regulating the initiation of sporulation in *Bacillus subtilis*. J Bacteriol *177*, 3308-3311.

6. Okamoto, S., Itoh, M., and Ochi, K. (1997). Molecular cloning and characterization of the obg gene of *Streptomyces griseus* in relation to the onset of morphological differentiation. J Bacteriol *179*, 170-179.

7. Okamoto, S., and Ochi, K. (1998). An essential GTP-binding protein functions as a regulator for differentiation in *Streptomyces coelicolor*. Mol Microbiol *30*, 107-119.

8. Verstraeten, N., Knapen, W.J., Kint, C.I., Liebens, V., Van den Bergh, B., Dewachter, L., Michiels, J.E., Fu, Q., David, C.C., Fierro, A.C., et al. (2015). Obg and Membrane Depolarization Are Part of a Microbial Bet-Hedging Strategy that Leads to Antibiotic Tolerance. Molecular cell *59*, 9-21.

9. Feng, B., Mandava, C.S., Guo, Q., Wang, J., Cao, W., Li, N., Zhang, Y., Wang, Z., Wu, J., Sanyal, S., et al. (2014). Structural and functional insights into the mode of action of a universally conserved Obg GTPase. PLoS Biol *12*, e1001866.

10. Murti, K.G., Webster, R.G., and Jones, I.M. (1988). Localization of RNA polymerases on influenza viral ribonucleoproteins by immunogold labeling. Virology *164*, 562-566.

11. Zielke, R., Sikora, A., Dutkiewicz, R., Wegrzyn, G., and Czyż, A. (2003). Involvement of the cgtA gene function in stimulation of DNA repair in *Escherichia coli* and *Vibrio harveyi*. Microbiology (Reading) *149*, 1763-1770.

12. Czyz, A., Zielke, R., Konopa, G., and Wegrzyn, G. (2001). A Vibrio harveyi insertional mutant in the cgtA (obg, yhbZ) gene, whose homologues are present in diverse organisms ranging from bacteria to humans and are essential genes in many bacterial species. Microbiology (Reading) *147*, 183-191.

13. Courcelle, J., Khodursky, A., Peter, B., Brown, P.O., and Hanawalt, P.C. (2001). Comparative gene expression profiles following UV exposure in wild-type and SOS-deficient *Escherichia coli*. Genetics *158*, 41-64.

14. Foti, J.J., Persky, N.S., Ferullo, D.J., and Lovett, S.T. (2007). Chromosome segregation control by Escherichia coli ObgE GTPase. Mol Microbiol *65*, 569-581.

15. Tan, J., Jakob, U., and Bardwell, J.C. (2002). Overexpression of two different GTPases rescues a null mutation in a heat-induced rRNA methyltransferase. J Bacteriol *184*, 2692-2698.

16. Du, S., Pichoff, S., and Lutkenhaus, J. (2016). FtsEX acts on FtsA to regulate divisome assembly and activity. Proc Natl Acad Sci U S A *113*, E5052-5061.

17. Xu, P., Ge, X., Chen, L., Wang, X., Dou, Y., Xu, J.Z., Patel, J.R., Stone, V., Trinh, M., Evans, K., et al. (2011). Genome-wide essential gene identification in *Streptococcus sanguinis*. Scientific reports *1*, 125.

18. Moraes, J.J., Stipp, R.N., Harth-Chu, E.N., Camargo, T.M., Höfling, J.F., and Mattos-Graner, R.O. (2014). Two-component system VicRK regulates functions associated with establishment of *Streptococcus sanguinis* in biofilms. Infect Immun *82*, 4941-4951.

19. Sham, L.T., Barendt, S.M., Kopecky, K.E., and Winkler, M.E. (2011). Essential PcsB putative peptidoglycan hydrolase interacts with the essential FtsXSpn cell division protein in *Streptococcus pneumoniae* D39. Proc Natl Acad Sci U S A *108*, E1061-1069.
